# Mutations in orthologous *PETALOSA* TOE-type genes cause a dominant double-flower phenotype in phylogenetically distant eudicots

**DOI:** 10.1093/jxb/eraa032

**Published:** 2020-01-21

**Authors:** Stefano Gattolin, Marco Cirilli, Stefania Chessa, Alessandra Stella, Daniele Bassi, Laura Rossini

**Affiliations:** 1 CNR-Consiglio Nazionale delle Ricerche, Istituto di Biologia e Biotecnologia Agraria (IBBA), Milano, Italy; 2 PTP Science Park, Lodi, Italy; 3 Università degli Studi di Milano – DiSAA, Milano, Italy; 4 Trinity College Dublin, Ireland

**Keywords:** *Dianthus*, double-flower, floral development, petal number, petunia, *Rosa*, tobacco

## Abstract

The double-flower phenotype has been selected by humans for its attractiveness in various plant species and it is of great commercial value for the ornamental market. In this study we investigated the genetic determinant of the dominant double-flower trait in carnation, petunia, and *Rosa rugosa*, and identified mutant alleles of TARGET OF EAT (TOE)-type genes characterized by a disruption of the miR172 target sequence and of the C-terminal portion of the encoded protein. Despite the phylogenetic distance between these eudicots, which diverged in the early Cretaceous, the orthologous genes carrying these mutations all belong to a single TOE-type subgroup, which we name as *PETALOSA* (*PET*). Homology searches allowed us to identify *PET* sequences in various other species. To confirm the results from naturally occurring mutations, we used CrispR-Cas9 to induce lesions within the miR172 target site of *Nicotiana tabacum PET* genes, and this resulted in the development of supernumerary petaloid structures. This study describes *pet* alleles in economically important ornamental species and provides evidence about the possibility of identifying and engineering *PET* genes to obtain the desirable double-flower trait in different plants.

## Teaser

A conserved mechanism regulates the dominantly inherited double-flower trait, and involves natural allelic variation at the microRNA172 binding site in the novel PETALOSA subclade of TARGET OF EAT (TOE)-type genes.

## Introduction

The variety of flower shapes and colours has fascinated humans for millennia, as witnessed by artistic depictions since ancient times. Among features of ornamental value, double-flower (DF) is a desirable trait that increases the number of petals and overall flower size (Crane and Lawrence, 1952). Investigations of the genetic control of this trait have uncovered different loci with recessive and dominant inheritance in several plant species (Vainstein, 2013). Recently, candidate mutations for the dominant DF trait have been described in orthologous genes of the euAP2 transcription factor lineage in peach (*Prunus persica*) and the *Rosa* genus (François *et al.*, 2018; Gattolin *et al.*, 2018; Hibrand Saint-Oyant *et al.*, 2018). euAP2 transcription factors are encoded by genes of the *APETALA2/ETHYLENE RESPONSIVE ELEMENT-BINDING FACTOR* (*AP2/ERF*) family and are divided into two groups, AP2-type and TARGET OF EAT (TOE)-type, both of which are characterized by the presence of a target site for the binding of microRNA172 (miR172), which negatively regulates their activity (Jofuku *et al.*, 1994; Riechmann *et al.*, 2000; Kim *et al.*, 2006). This class of transcription factors play a conserved role in the regulation of flower patterning and development transitions (Huijser and Schmid, 2011), with AP2 playing a major role in the ABCDE model proposed for Arabidopsis (Rijpkema *et al.*, 2010). During flower development, repression of euAP2 by miR172 is crucial for maintaining organ determinacy and for defining the boundary between the outer sterile perianth and the reproductive inner flower parts (Zhao *et al.*, 2007; Wollmann *et al.*, 2010). Wild-type Rosaceae flowers typically bear five petals. In both peach and rose, mutated alleles in orthologous TOE-type genes are dominantly associated with the DF phenotype, suggesting they might act as upstream regulators of AGAMOUS (AG) and restrict its expression to the inner floral whorls (Dubois *et al.*, 2010): such variants cause the expression of transcripts encoding truncated proteins that carry all the key functional motifs but lack the miR172 target site. In particular, a deletion in the 3´ region of the peach gene *Prupe.6G242400* results in the expression of a truncated mRNA, and ultimately in a potentially functional TOE-type transcription factor that is not post-transcriptionally regulated by miR172 (Gattolin *et al.*, 2018). In rose, an insertion in the 8th intron of *RcHm3g0468481*, encoding the TOE-type factor XP_024182693, causes a premature stop that also leads to the transcription of a truncated mRNA lacking the miR172 binding site (Gattolin *et al.*, 2018). The insertion in rose seems to consist of a Gypsy long terminal repeat (LTR) transposon and this allele can be found in the ‘Old Blush’ rose genome, suggesting that this variant could have originated in *Rosa chinensis*, a major contributor to the genetic make-up of modern roses (Martin *et al.*, 2001; Bendahmane *et al.*, 2013; François *et al.*, 2018). However, previous research has suggested that different mutations leading to the DF phenotype have occurred in the genus and were probably selected independently in *R. chinensi*s, *R. gallica*, and *R. rugosa* (Dubois *et al.*, 2010).

Dominant flower doubleness is widespread amongst angiosperms, and it has been selected as a leading ornamental trait in commercially important plants such as carnation (*D85* locus; Yagi *et al.*, 2014*a*) and petunia (*Do1* locus; Sink 1984; de Vlaming *et al.*, 1984; Van Der Krol and Chua, 1993). However, the genes controlling this phenotype remain unknown in these species. In this study, we investigated the genetic determinant of the dominant DF trait in carnation, petunia, and *R. rugosa*, and discovered mutant alleles of TOE-type genes—hereafter referred to as *PETALOSA* (*PET*) genes—characterized by a disruption of the miR172 target sequence. To further support a conserved role of allelic variability in *PET* genes, we used a genome-editing approach to induce mutations within the miR172 target site of *Nicotiana tabacum PET* genes and obtained tobacco lines characterized by additional petals and petaloid stamens.

## Materials and methods

### Plant material

*Dianthus* varieties were kindly provided by Floricoltura Billo (https://www.floricolturabillo.it/) and Hybrida srl. *Rosa rugosa* material was kindly provided by Le Rose di Firenze (https://www.lerosedifirenze.com). *Petunia* plants were obtained from local garden centres and are identified using their commercial names.

### RT-PCR and 3´-RACE

Total RNA was extracted from floral buds using a Quick-RNA Miniprep Kit (Zymo) following the manufacturer’s protocol, with the modification of adding 2% PVP and 4% beta-mercaptoethanol (Sigma-Aldrich) to the tissue lysis buffer just before use. Then, 1 μg total RNA was treated with DNAseI (Invitrogen) and first-strand cDNA was obtained using Goscript Reverse Transcriptase (Promega), using either a standard oligo-dT primer or the B26 primer containing an adaptor sequence (Frohman *et al.*, 1988). Reactions were diluted 1:10 and 1 µl was used as the template for RT-PCR analysis or for 3´-RACE (rapid amplification of cDNA ends) using GOTaq (Promega). RT-PCR analysis was carried out in a 25-µl reaction with GOTaq, using the specific primers CA-9f/CA-1r (*Dianthus pet* allele), CA-9f/CA-4r (*Dianthus* wild-type allele), PH-9F/PH-M-3R (petunia *pet* allele), or PH-9F/PH-10R (petunia wild-type allele). RACE analysis was carried out in a 25-µl reaction using GOTaq from double-flower (DF) petunia cDNA using PH-9F and B25 or from DF *Dianthus* cDNA using CA-9f and B25.

### Genome-walking in *R. rugosa*

Samples of 2 µg of single-flower and DF *R. rugosa* genomic DNA were digested with 2 µl TaqIa (NEB) in Buffer Cutsmart in a 20-µl final reaction (65 °C, 2.5 h). The digestion reaction was purified with PCR clean-up kit (Promega) and the concentration adjusted to 50 ng µl^–1^. Then, 20 µl of B25 and B25_TaqI_adapter primers (100 pmol µl^–1^) were mixed, heated for 5 min at 75 °C and allowed to cool down to room temperature to anneal into 5´-CG-3′ overhang adapters. Following this, 200 ng of digested DNA and 100 pmol of B25 adapter were ligated using T4 Ligase (Invitrogen). The ligation was used as template for a two-step nested PCR. A 13-cycle pre-PCR cycle was carried out using RO-8F/B25, and this was used as the template for nested PCR, using RO-8F2/B25. Amplified bands were gel-purified and sequenced.

### Genotyping

Genomic DNA was extracted from 200 μg of leaf tissue using a DNeasy 96 Plant Kit (Qiagen); 10 ng samples of genomic DNA were used in the PCR reactions using GoTaq and the appropriate primer combinations (Supplementary Table S1 at *JXB* online) as follows: CA-9f/CA-4r or CA-9f/CA-1r, (*Dianthus* wild-type and *pet* alleles, respectively); PH-9F/PH-10R or PH-9F/PH-M-3R (*Petunia hybrida* wild-type and *pet* alleles, respectively); and RO-8f/RR-3UTRr for both *R. rugosa* wild-type and *pet* alleles.

### Full-length genomic DNA sequencing

Full-length genomic amplicons were obtained with GoTaq Long (Promega) using genomic DNA obtained from *D. barbatus* ‘Sweet William’, *D. superbus* ‘Primadonna’, *D. caryophyllus* ‘Widecombe fair’, *P. hybrida* ‘Viva Double Purple Vein’ (Florensis), *R. rugosa* ‘Hansa’, using the primers CA-F1/CA-4R (*Dianthus*), PH-F1/PH-UTR-R (petunia wild-type allele), PH-F1/PH-M-3R (petunia *pet* allele), and RO-1F/RR-UTR-R (*R. rugosa*). PCR bands were extracted from agarose gels using a Wizard SV Gel and PCR Clean-Up System (Promega) and paired-end sequenced on an Illumina MiSeq instrument, following the manufacturer’s instructions, using a transposome-based Nextera XT kit (Illumina) to generate the libraries. FASTQ files were mapped with BWA-MEM against reference sequences on the Galaxy Platform (Afgan *et al.*, 2018) and visualized using IGV (Robinson *et al.*, 2011).

### Molecular phylogenetic analysis by maximum-likelihood method

Peptide sequences used for phylogenetic analysis from *P. persica*, *R. chinensis*, *P. hybrida*, *P. axillaris*, *A. thaliana*, and *Vitis vinifera* were obtained from previously published work (Morel *et al.*, 2017; Gattolin *et al.*, 2018), sequences from *Nicotiana tomentosiformis*, *Camellia sinensis*, *Spinacia oleracea*, and *Carica papaya* were obtained from the NCBI database (https://www.ncbi.nlm.nih.gov/), and sequences for *D. caryophyllus* were obtained from the reference genome website (http://carnation.kazusa.or.jp/). For *Dianthus* Dca21030 the wild-type allele (Dca21030.2) was used (see Results). Phylogenetic relationships were estimated in MEGAX (Kumar *et al.*, 2018). Peptide sequences (Supplementary Fig. S1A) were aligned by MUSCLE with default settings. Evolutionary relationships among TOE-type members were inferred by using the maximum-likelihood method based on the JTT matrix-based model. The rate variation model allowed for some sites to be evolutionarily invariable and a discrete Gamma distribution was used to model evolutionary rate differences among sites. The reliability of the phylogenetic tree was estimated by setting 200 bootstrap replicates.

### CrispR-Cas9 editing of tobacco

For genome editing of tobacco plants, we used *Agrobacterium*‐mediated T‐DNA transformation with the binary vector pHAtC (Kim *et al.*, 2016), obtained from Addgene (https://www.addgene.org). To ensure the transcription of specific guide RNA, the oligonucleotides TOB-CRISPR_for and TOB-CRISPR_rev were annealed and ligated into *AarI*-digested pHAtC. *Nicotiana tabacum* cv TI 527 ‘Kentucky’ plants were transformed with a c58 *Agrobacterium* suspension following the method of Sparkes *et al.* (2006) and transformants were selected in media containing Hygromycin (30 mg l^–1^). T_0_ plants were grown under standard greenhouse conditions until flowering. Mutations in the *NtBEN* miR172 binding sites were assessed by Sanger sequencing of PCR fragments obtained using either the forward primer NtBEN_016482517_F (XP_016482517) or NtBEN_016499635_F (XP_016499635), and a common reverse primer, NtBEN_SEQR. T_1_ seedlings were PCR-screened for the presence of the transgene using the primers LBfor/LBrev and RBfor/RBrev, specific for the T-DNA sequence. The presence of off-targets in transgene-free T_1_ plants was assessed using a high-resolution melting (HRM) analysis-based approach. Primers (Supplementary Table S1) were designed to amplify fragments flanking the PAM recognition sequence within the miR172 target site of the euAP2 target genes and HRM analyses were carried out in a Corbett Rotor-Gene 6000 series using a Type-it HRM PCR Kit (both Qiagen). The reactions were carried out with the following program: 2 min at 94 °C, 40 cycles of 30 s at 94 °C, 30 s annealing at 58 °C and 30 s at 72 °C, followed by a melting step over a 70–95 °C gradient with 0.1 °C s^–1^ ramp rate. Data were analysed using the Rotor-Gene software 1.7 and visualized using both a derivative and difference plot, according to the software instructions.

## Results

### A mutation disrupting the miR172 target site of a *PET* gene is associated with the DF phenotype in *Dianthus*

**Fig. 1.**
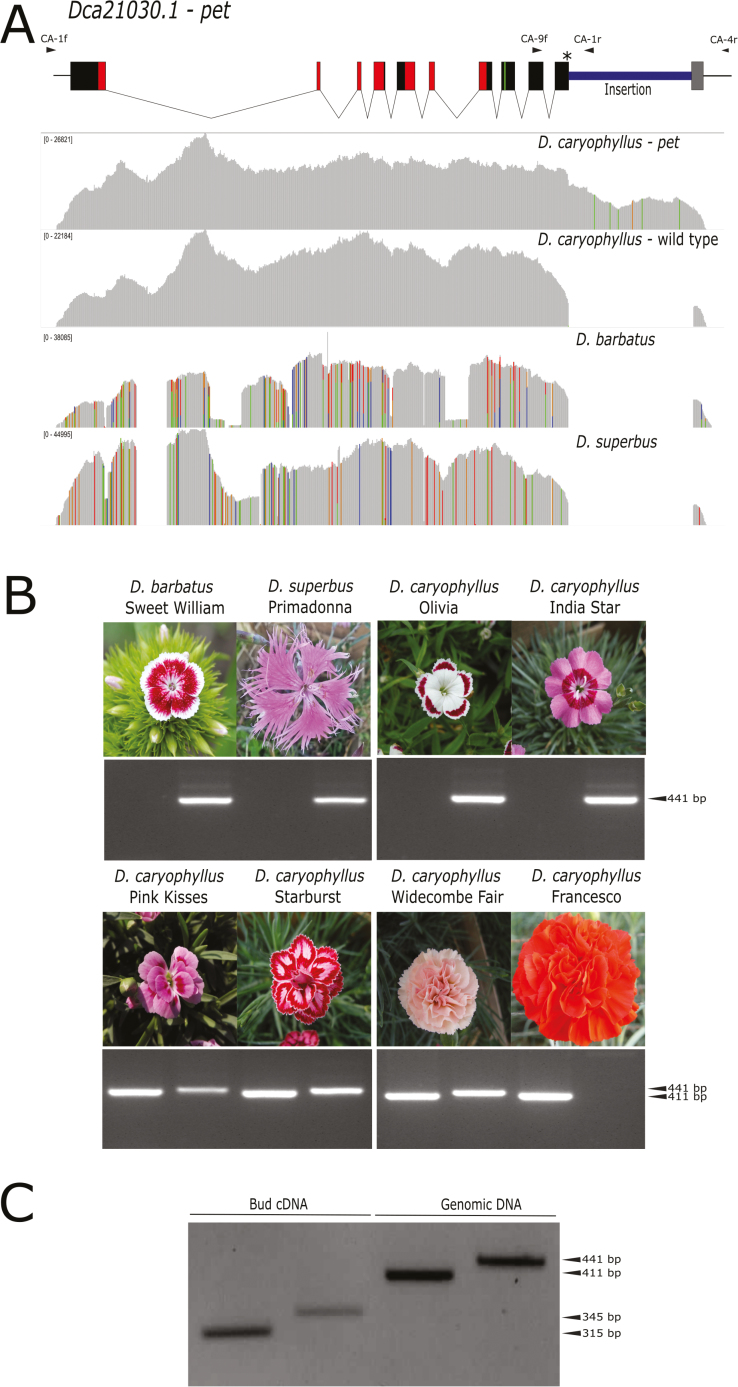
Molecular analysis of sequence variants of the *Dianthus PETALOSA* (*PET*) gene. (A, top) Model of *Dca21030.1* (*Dianthus pet* allele). Solid boxes indicate the CDS exons, arrowheads indicate the positions of the primers CA-1f, CA-9f, CA-1r, and CA-4r, and the asterisk indicates the miR172 target region. The coding sequences for the AP2-R1/AP2-R2 conserved domains and the EAR motif are marked in red and green, respectively. (A, bottom) Amplicon-sequencing coverage plot of the two alleles amplified with the primers CA-1f and CA-4r from the double-flower *D. caryophyllus* ‘Widecombe Fair’ and of the single amplicon obtained from both *D. barbatus* and *D. superbus*. (B) PCR analysis of four single-flower (top) and four double-flower (bottom) *Dianthus* accessions. The expected band sizes for *pet* (CA-9f/CA-1r, 411bp) and the wild-type allele (CA-9f/CA-4r, 441bp) are indicated. (C) Amplification with primers the CA-9f/CA-1r and CA-9f/CA-4r of flower bud cDNA (*pet*, 315 bp; wild-type, 345bp) and genomic DNA of *D. caryophyllus* ‘Widecombe Fair’.

The *Dianthus* genus comprises species of horticultural interest (carnations and pinks) that include both single- and double-flower varieties. Homology searches on the carnation (*Dianthus caryophyllus* L.) genome (Yagi *et al.*, 2014*b*) allowed the identification of a TOE-type gene orthologous to *Prupe.6G242400*, annotated as *Dca21030.1*. Notably, *CES0212*, an SSR marker tightly associated with the DF *D85* locus (Yagi, 2014*a*), was mapped less than 5 kb from the 5´ region of *Dca21030.1*, making this gene a prime candidate for the DF phenotype. Analysis of the reference genome sequence of the DF cultivar ‘Francesco’ revealed that this gene consisted of 10 exons. Sequence comparison with peach *Prupe.6G242400* suggested the presence of a 1-kb insertion within the 10th exon (Supplementary Fig. S2A), also affecting the miR172 target site. The sequence of the insertion showed similarity with a putative mobile element present in multiple copies within the *D. caryophyllus* genome (Supplementary Fig. S2B), and we reasoned that this annotated gene could be the mutated *PET* allele (*pet*) that leads to flower doubleness in ‘Francesco’. Allelic comparison of the 3´ region revealed the presence of only the wild-type allele in the single-flower accessions *D. superbus* ‘Primadonna’ and *D. deltoides* ‘Flashing lights’ (Fig. 1A, B, Supplementary Fig. S2C) and both the wild-type and *pet* alleles in the DF variety ‘Widecombe fair’ (Fig. 1A). Supporting the role of these sequence variants in flower development, both alleles were expressed in ‘Widecombe fair’ floral buds (Fig. 1C). Combined evidence from cDNA and genomic resequencing confirmed the presence of a 1-kb insertion, causing a CC to AG substitution at the 3´ end of the miRNA target site and introducing a stop codon 11 bp downstream (Supplementary Fig. S2C, D), consistent with the annotated genome sequence. Therefore, *Dca21030.1* could represent a *de facto pet* allele encoding a transcript that escapes regulation by miR172 and confers the DF phenotype. Conversely, the identified wild-type allele (*Dca21030.2*) harboured a complete miR172 target site and a further 83 bp of coding sequence (Supplementary Fig. S3A, B). Co-segregation of the *pet* allele with the dominant DF phenotype was confirmed in 25 commercial varieties (Fig. 1B, Supplementary Table S2A).

### A mutation disrupting the miR172 target site of a *PET* gene is associated with the DF phenotype in petunia

**Fig. 2.**
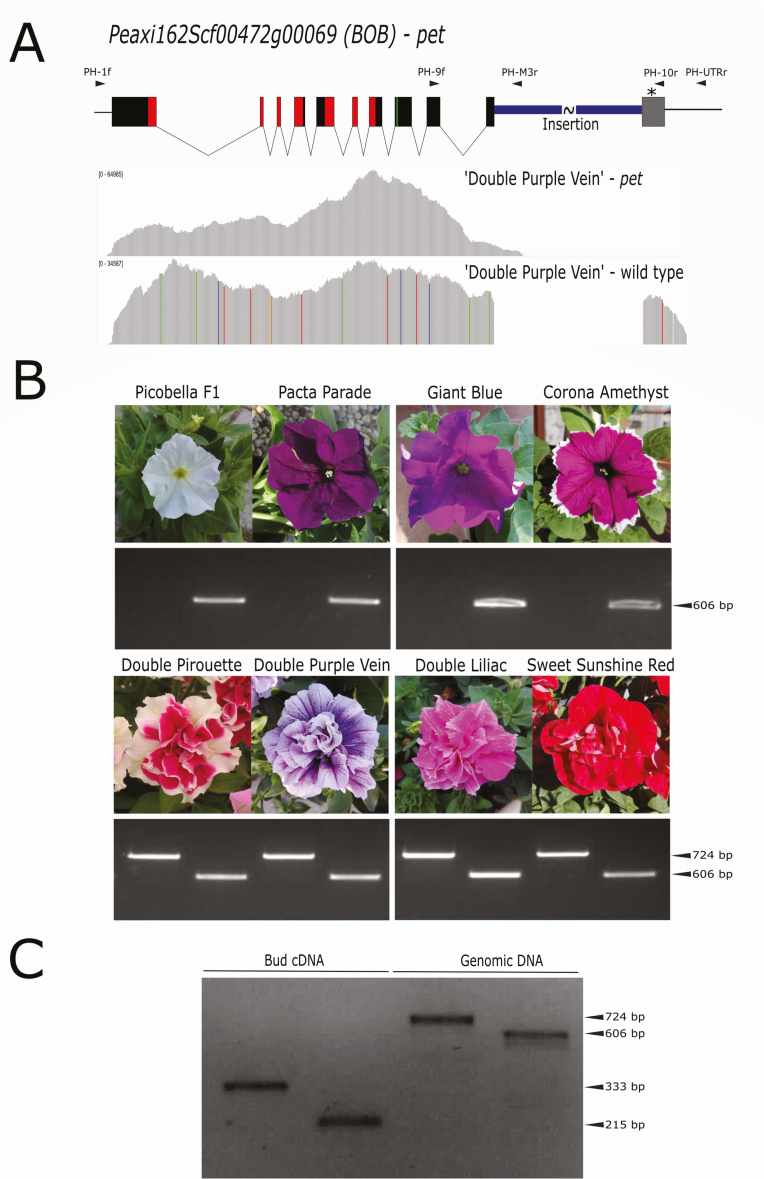
Molecular analysis of sequence variants of the petunia *PETALOSA* (*PET*) gene. (A, top) Model of the petunia *BOB pet* allele deduced from the genomic sequence of *Peaxi162Scf00472g00069* and the available sequence information for the insertion. Solid boxes indicate the CDS exons, arrowheads indicate the positions of the primers PH-1f, PH-9f, PH-10r, PH-M3r, and PH-UTRr, and the asterisk indicates the miR172 binding site. The coding sequences for AP2-R1/AP2-R2 conserved domains and the EAR motif are marked in red and green, respectively. (A, bottom) Amplicon sequencing coverage plot of the two alleles amplified from the double-flower petunia ‘Double Purple Vein’ with the primers PH-1F and PH-M3r (*pet*) or PH-UTRr (wild-type). (B) PCR analysis of four single-flower (top) and four double-flower (bottom) petunia varieties. Expected band sizes for the *pet* (PH-9F/PH-M-3R, 724 bp) and the wild-type allele (PH-9F/PH-10R, 606 bp) are indicated. (C) PCR products obtained with the primers PH-9F/PH-M-3R and PH-9F/PH-10R using flower bud cDNA (*pet*, 333 bp; wild-type, 215 bp) or genomic DNA of the petunia variety ‘Double Purple Vein’. (Photograph of ‘Double Pirouette’ by 阿橋 HQ, https://www.flickr.com/photos/nhq9801/9252404839/; CC BY-SA 2.0, https://creativecommons.org/licenses/by-sa/2.0.)

BLIND ENHANCER (BEN) and BROTHER OF BEN (BOB), two *Petunia hybrida* TOE-type transcription factors orthologous to Prupe.6G242400, have been finely characterized and are suggested to redundantly regulate the development of the second and third floral whorls (Morel *et al.*, 2017). The sequence of the genomic marker SSR7 associated with the DF phenotype in petunia (Liu *et al.*, 2016) was searched against the genomes of the wild parents, *P. axillaris* and *P. inflata* (Bombarely *et al.*, 2016): this analysis positioned the marker within the *P. inflata* scaffold Peinf101Scf00457, at 1310 kb from the *BOB* genomic sequence. The possible involvement of this gene in the *P. hybrida* DF phenotype was investigated by 3´-RACE on bud cDNA from the DF variety ‘Double Purple Vein’. Of the two expressed alleles obtained, one was nearly identical to the *BOB* transcript (KU096996) and *P. axillaris Peaxi162Scf00472g00069.1*, while the second showed an insertion positioned at the level of the 10th exon, 69 bp upstream of the miR172 target site (Fig. 2A, Supplementary Fig. S4A), as confirmed by targeted resequencing. The insertion—a probable LTR mobile element present in multiple copies in the genomes of *P. axillaris* (Supplementary Fig. S4B, C) and *P. inflata*—resulted in a shorter transcript that was predicted to escape miR172 post-transcriptional regulation whilst still encoding euAP2 functional domains (Supplementary Fig. S3C, D). The genotype–phenotype association was validated in different commercial petunia varieties (Fig. 2B). Interestingly, in the four DF varieties tested, both the wild-type and the *pet* allele were found, in agreement with previous reports on the presence of both alleles in DF petunia varieties (Sink, 1984). Expression of both alleles was further confirmed in ‘Double Purple Vein’ using specific primers (Fig. 2C).

### Identification of a previously uncharacterized *pet* allele in *R. rugosa*

**Fig. 3.**
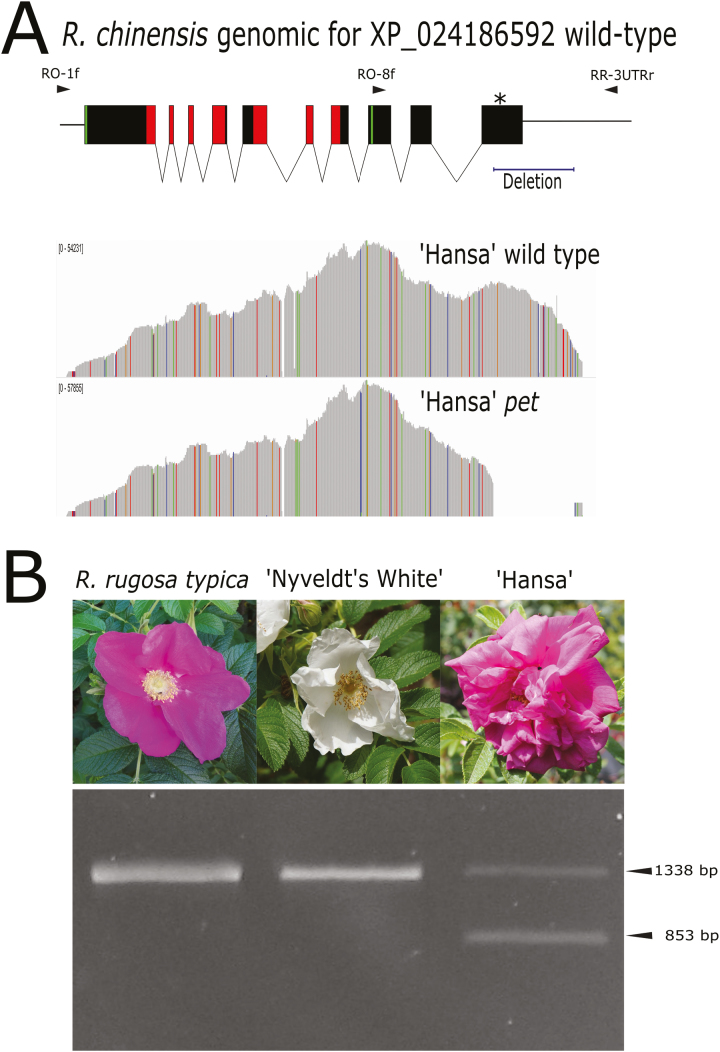
Molecular analysis of sequence variants of the *Rosa rugosa PETALOSA* (*PET*) gene. (A, top) Model of *R. chinensis* wild-type *PET* (*XP_024186592*). Solid boxes indicate the CDS exons, arrowheads indicate the positions of the primers RO-1f, RO-8f, and RR-UTRr, and the asterisk indicates the miR172 binding site. The coding sequences for the AP2-R1/AP2-R2 conserved domains and the EAR motifs are marked in red and green, respectively. (A, bottom) Amplicon sequencing coverage plot of the two alleles amplified with the primers RO-1f and RR-UTRr from the double-flowered *R. rugosa* ‘Hansa’. (B) PCR analysis of two single-flower accessions and DF ‘Hansa’ using the primers RO-8f e RR-3UTRr. Expected band sizes for the *pet* (853 bp) and the wild-type allele (1338 bp) are indicated.

The screening of different rose varieties with the previously developed *R. hybrida pet* marker (Gattolin *et al.*, 2018) only detected the wild-type allele in the DF *R. rugosa* variety ‘Hansa’. Considering the complex breeding history of modern roses (Dubois *et al.*, 2010), we hypothesized the existence of a different but functionally similar *pet* allele in *R. rugosa*. A genome-walking approach revealed a 500-bp deletion in the *R. rugosa* gene orthologous to *RcHm3g0468481*, spanning part of the last exon and the adjacent 3´ UTR (Fig. 3A). The deletion included the miR172 binding sequence, and resulted in a *pet* allele similar to those previously identified in the other DF species but not found in single-flower *R. rugosa* accessions (Fig. 3B). Targeted resequencing of the entire gene regions of both ‘Hansa’ alleles, using the wild-type *R. chinensis* allele as the reference, confirmed the presence of the deletion in this *pet* allele (Fig. 3A, Supplementary Fig. S3E–G). The presence of this *pet* allele was further confirmed in four other commercial DF varieties (Supplementary Table S2B), indicating that in the *Rosa* genus the DF phenotype can be caused by at least two independent *pet* mutations.

### Phylogenetic analysis of TOE-type transcription factors

**Fig. 4.**
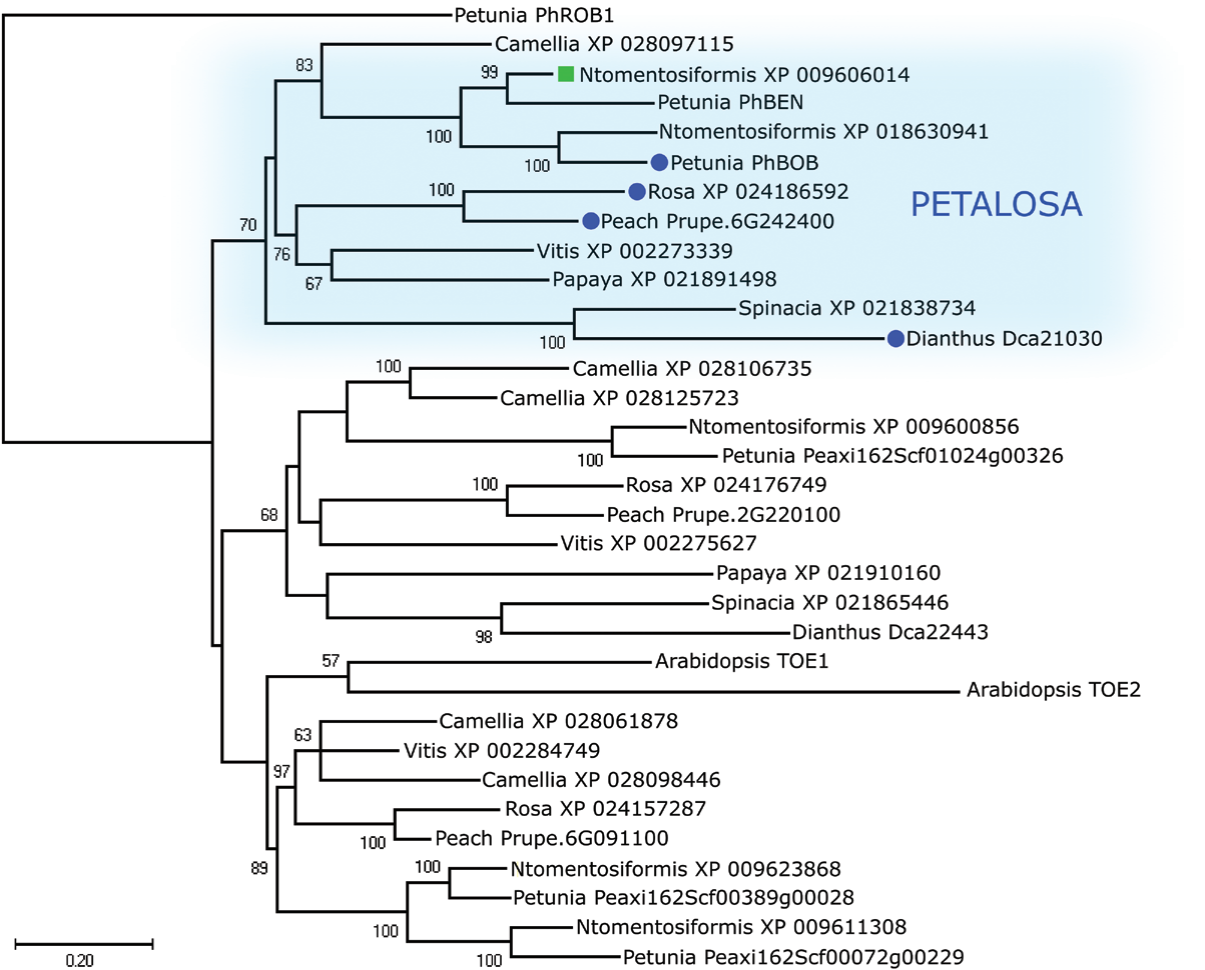
Evolutionary relationships among TOE-type proteins in different plant species. The tree was obtained using peptide sequences from *Prunus persica* (peach), *Rosa chinensis*, *Arabidopsis thaliana*, *Petunia hybrida*/*axillaris*, *Dainthus caryophyllus*, *Spinacia oleracea*, *Nicotiana tomentosiformis* (Ntomentosiformis), *Vitis vinifera*, *Carica papaya*, and *Camellia sinensis*. The circles indicate characterized PETALOSA genes and the square indicates *N. tomentosiformis* BEN. The *R. chinensis* sequence XP_024186592 corresponds to RAG04722 described previously by Gattolin *et al.* (2018). An AP2-type sequence from petunia (PhROB1) was included as the outgroup. Only bootstrap values >50 are shown. (This figure is available in colour at *JXB* online.)

To gain insights into the phylogenetic relationships among TOE-type genes harbouring *pet* mutations, we analysed protein sequences from different plant species (listed in Supplementary Fig. S1A). As the Rosaceae family was represented by sequences from both rose and peach, sequences from a second Solanaceae species, diploid *N. tomentosiformis*, were included to complement those of petunia. Phylogenetic analysis suggested the existence of three subgroups within the TOE-type genes (Fig. 4), and all genes associated with DF mutations belonged to a single orthologous *PET* subgroup, which included a gene/genomic duplication in *N. tomentosiformis* as well as petunia, where this is consistent with functional redundancy (Morel *et al.*, 2017). Hence, independent selection of distinct mutations in orthologous *PET* genes gave rise to the DF trait in peach, rose, carnation, and petunia. The lack of a *PET* gene in Arabidopsis could be due to a recent loss following polyploidization and genome rearrangements in the Brassicaceae lineage (Blanc *et al.*, 2003; Town *et al.*, 2006; Ren *et al.*, 2018), and a survey of TOE-type genes (Supplementary Fig. S1B) confirmed the absence of *PET* genes in *Brassica napus*. Interestingly, a *PET* orthologue (XP_021891498) was found in *C. papaya*, which belongs to the order Brassicales, suggesting that a *PET* was indeed originally present in the lineage. A survey of the reference allotetraploid *N. tabacum* genome (Murad *et al.*, 2002) revealed the existence of at least 13 putative euAP2 proteins, including three putative PET sequences, namely the two closely related homoeologues NtBENa and NtBENb (XP_016482517 and XP_016499635, derived from *N. tomentosiformis* and *N. sylvestris*, respectively), and a NtBOB (XP_016502850, derived from *N. tomentosiformis*, XP_018630941) (Supplementary Fig. S1C, D). Notably, in both *N. tabacum* and *N. tomentosiformis* BOB transcripts, a miR172 binding site was present in the 3´ UTR, while a stop codon positioned 14 nucleotides upstream of the site itself (Supplementary Fig. S5A–C) resulted in a predicted protein lacking 54 amino acids at the C-terminus, compared to petunia BOB (Supplementary Fig. S5D).

### Validation of the effect of lesions in the miR172 binding site in *PET* genes via CrispR-Cas9 editing of tobacco plants

**Fig. 5.**
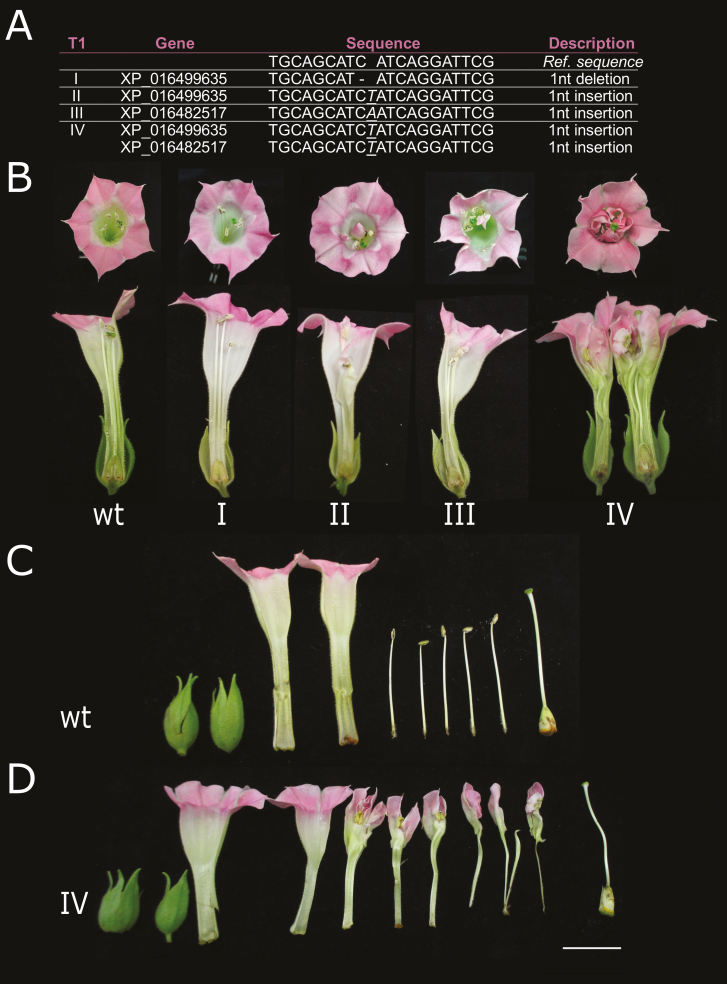
Gene-editing of tobacco (*Nicotiana tabacum*) plants by CrispR-Cas9. (A) Heterozygous mutations at *NtBEN* miR172 target sites in four T_1_ genome-edited tobacco lines, I–IV. *XP_016482517* is *NtBENa* and *XP_016499635* is *NtBENb* (Supplementary Fig. S1D). (B) Flower phenotypes of a wild-type (wt) and the four T_1_ plants. (C) Dissection of a wild-type flower showing sepals, fused petals, stamens, and pistil. (D) Dissection of a T_1_ line IV flower showing sepals, fused petals, petaloid stamens, and pistil. The scale bar is 1 cm. (This figure is available in colour at *JXB* online.)

Our results demonstrated a strict association between a range of naturally occurring mutations in orthologous *PET* genes and the dominant DF trait in different plant species. We therefore investigated whether artificially induced mutations in the miR172 target site of a *PET* gene would be sufficient to induce the DF phenotype in a different plant species without naturally occurring DF variants. To this end, we used CrispR-Cas9 to specifically create mutations at the *PET*-miR172 target sequence in tobacco (Supplementary Fig. S5E). Of seven T_0_ tobacco lines carrying the CRISPR-Cas9 construct, three were characterized by a range of floral phenotypes that included conversion of stamens into petaloid structures and double flowers (Supplementary Fig. S6). Molecular analyses of leaf and petal tissues confirmed an array of mutations, ranging from 1–3-bp insertions and/or deletions in the miR172 binding site of target *NtBEN*s and *NtBOB*, while the other euAP2s were not affected by the editing (Supplementary Fig. S7). Thus, the preliminary T_0_ analysis confirmed that plants displaying the DF phenotype also carried lesions in the *PET* miR172 binding regions. As a result of selfing T_0_ line 7, four T_1_ plants lacking the CRISPR-Cas9 construct and carrying mutations in *NtBEN*s were selected, while no plants with a mutation in *NtBOB* were obtained (Supplementary Fig. S7). Sequencing of genomic fragments spanning the miR172 target site of both *NtBEN*s revealed the presence of differently edited alleles, associated in heterozygosity to various degree of flower doubleness (Fig. 5). A single-nucleotide insertion within the miR172 core recognition sequence of either *NtBEN* gene was sufficient to induce the development of petaloid structures within the corolla (Fig. 5, lines II and III). A 1-bp deletion in one of the two *NtBEN* genes resulted in flowers that were indistinguishable from the wild-type (line I), while a 1-bp insertion in both *NtBEN* genes resulted in the strongest DF phenotype (line IV).

## Discussion

The understanding of the mechanisms governing flower development has been a major goal in plant biology, and knowledge gained from model species does not always apply to other plants. Initial insights into the molecular basis of dominant mutations that confer the double-flower (DF) trait were recently obtained when structural variants that disrupt miR172 target sites within a subclass of TOE-type genes were pinpointed as prime candidates in Rosaceae (François *et al.*, 2018; Gattolin *et al.*, 2018), although functional evidence remained lacking. Whether the mechanism behind the dominant DF trait is conserved in phylogenetically distant genera of angiosperms has also remained unknown and is of great interest for both plant scientists and breeders, particularly considering the economic value of the ornamental flower market.

In the present study, we show that similar variations may be responsible for the dominant DF character in different plants, thus broadening the spectrum of species bearing this type of mutation beyond the Rosaceae family. Through allele-mining approaches, *pet* mutations in orthologous genes encoding PET euAP2 transcription factors were also identified in carnation, petunia, and *R. rugosa*. Insertions in carnation and petunia and a deletion in *R. rugosa* were shown to result in the transcription of mRNAs encoding truncated proteins lacking the C-terminus portion and disrupting the miR172 target site within (Figs 1–3, Supplementary Fig. S3). By editing the genome of a single-flower species (tobacco), we clearly demonstrated the crucial role of *PET* genes in the DF phenotype: single-nucleotide insertions within the miR172 sequence core induced the development of supernumerary petaloid stamens in whorl 3 (Fig. 5). In contrast to the strong reduction in petal development caused by double-knockout of the petunia *PET* genes *BEN* and *BOB* (Morel *et al.*, 2017), *pet* alleles are dominantly associated with supernumerary petals, indicating that they are gain-of-function mutations. In agreement with the well-characterized mechanism of miR172-regulated euAP2 expression, all these *pet* alleles are likely to escape post-transcriptional regulation. This might lead to the DF phenotype as a consequence of altered repression of AG, similar to AG regulation by AP2 in Arabidopsis (Krogan *et al.*, 2012; Morel *et al.*, 2017).

As *pet* mutations also affect the C-terminal portion of the encoded transcription factors (Supplementary Fig. S3), the detailed mechanism by which naturally occurring or artificially induced mutations modify the functionality of *PET* genes and give rise to the DF trait remains to be more precisely validated. Nevertheless, different lines of evidence support a crucial role for the miR172 binding site rather than modification of protein C-terminal functionality. First, all the identified *pet* alleles still encoded all the highly conserved functional euAP2 domains (Supplementary Fig. S3; Wang *et al.*, 2016), while the C-terminal portions of the PET proteins showed little conservation beyond the motif corresponding to the miR172 binding site (Supplementary Fig. S8A). Indeed, *BOB* genes in wild-type single-flower *N. tabacum* and *N. tomentosiformis* encoded shorter PET proteins and harboured the miR172 binding sequence in the 3´ UTR (Supplementary Figs S5A, S8B). Second, in cases where the miR172 target site was affected but not completely absent, the severity of the phenotype depended on the position of the mutation within the site (Fig. 5). This can be interpreted as the result of a different stability of the resulting mRNA:miRNA duplexes and a consequent moderate loss of *PET* regulation, lowering but not abolishing AG expression. In *Dianthus pet* we observed two consecutive mismatches corresponding to the miRNA 5´ end (Supplementary Fig. S3A), which has been reported to fully abolish target site efficacy (Liu *et al.*, 2014). Furthermore, mRNA regions flanking the miR172 binding site have also been shown to affect silencing efficiency (Li *et al.*, 2014; Wong *et al.*, 2018) and the different downstream sequence may well contribute to the phenotype severity in *Dianthus*. Third, the tobacco T_1_ plant with the strongest phenotype (line IV; Fig. 5) harboured a 1-bp heterozygous insertion in both *NtBEN* genes, suggesting a dose-dependent effect. This is reminiscent of previous observations of the *AP2*-like *Q* gene in polyploid wheat: a single-nucleotide mutation in the miR172 target site was shown to alter the balance between miR172 and *Q* gene expression and to correlate with dose-dependent phenotypes of varying intensity (Debernardi *et al.*, 2017).

In the present study, we identified the PET subgroup of TOE-type euAP2 transcription factors comprising sequences from eudicot species belonging to the Pentapetalae clade (Fig. 4), a group of plants characterized by pentamerous flowers with whorled phyllotaxis. Pentapetalae are divided in two groups (Zeng *et al.*, 2017) and *PET* sequences were found both in Rosids (peach, rose, papaya) and Vitales (*V. vinifera*) belonging to Group I, as well as in Asterids (petunia, *Nicotiana*, camellia) and Caryophyllales (*Dianthus*, *Spinacia*) belonging to Group II. These two groups are estimated to have diverged around 120 Mya, in agreement with the radiation of the Pentapetalae lineage that recent studies have set in the early Cretaceous (Kumar *et al.*, 2017; Zeng *et al.*, 2017). Given that the single-flower phenotype is the ancestral state of peach, rose, carnation, and petunia, and that independent mutations were found in orthologous *PET* genes, the dominant DF trait probably represents an example of convergence under strong human selection in phylogenetically distant eudicots. Arabidopsis TOE-type genes (*TOE1* and *TOE2*) have been reported to act redundantly in various plant developmental processes, such as seedling innate immunity (Zou *et al.*, 2018), epidermal leaf identity (Wu *et al.*, 2009), and repression of flowering (Aukerman and Sakai, 2003; Jung *et al.*, 2007; Zhai *et al.*, 2015; Zhang *et al.*, 2015). While our phylogenetic reconstruction placed these proteins close to one of the non-PET TOE-type subgroups (Fig. 4), this positioning was not strongly supported (bootstrap value 47): in conjunction with the long branches, this suggests an uncertain phylogenetic placement. These considerations and the lack of a PET orthologue indicate that Arabidopsis may not be representative of the functions of TOE-type genes in other species. A question that remains outstanding is the role of genes from the other two TOE-type subgroups. Further studies in a range of plants will therefore be important to gain a better understanding of the biological roles of different TOE-type genes.

In summary, we used information on the causal mutation of the dominant DF phenotype in peach and rose to investigate other economically important ornamental plants, namely carnation, petunia, and *R. rugosa*. In all cases, we identified strong candidate causal mutations in orthologous TOE-type genes that resembled those previously described. These findings were confirmed by phenotypic alterations in gene-edited tobacco plants, providing a proof-of-concept of the possibility to manipulate flower morphology in different plants through *PET* engineering.

## Supplementary data

Supplementary data are available at *JXB* online.

Fig. S1. TOE-type peptides from different species.

Fig. S2. Analysis of the *Dianthus pet* allele.

Fig. S3. Sequence alignment of *PET* alleles from different species.

Fig. S4. Analysis of the petunia *pet* allele.

Fig. S5. Analysis of the tobacco *PET* alleles

Fig. S6. T_0_ tobacco lines transformed with the CrispR-Cas9 construct.

Fig. S7. High-resolution melting analysis for detection of potential off-target edited alleles of tobacco TOE-type genes.

Fig. S8. Degree of conservation of PET amino acid sequences and effects of CrispR-Cas9-induced mutations on protein sequences.

Table S1. List of primers used in this study.

Table S2. PCR analyses of *Dianthus* and *R. rugosa* varieties.

eraa032_suppl_Supplementary_Figures_S1-S8_Tables_S1-S2

## Data availability

The FASTQ files of the amplicon sequencing data have been deposited at the NCBI Sequence Read Archive (SRA; https://www.ncbi.nlm.nih.gov/sra) under BioProject accession number PRJNA600422.
