# Supplementary material for: Mutations in orthologous PETALOSA TOE-type genes cause a dominant double-flower phenotype in phylogenetically distant eudicots
Source: J Exp Bot. 2020 Jan 21;71(9):2585–95. doi: 10.1093/jxb/eraa032 (PMC7210751; doi:10.1093/jxb/eraa032)
Supplement: eraa032_suppl_Supplementary_Figures_S1-S8_Tables_S1-S2 [file eraa032_suppl_supplementary_figures_s1-s8_tables_s1-s2.pdf]

Figure S1

A

>Petunia_PhROB1

MWDLNDSPDQRREINIDESEEGCSSHIELEPDDEKGKRVGSFSTSSSSAIAIDEISEEEDGEKGKKKRSSPSKLFGFSMVGPGDLEQPITR
QFFPVDEAEAETGVVTNGSLNFPRAHWVGVKFYQNEPLGITGVVDVTQQQQQQQPMKKSRRGPRSRSSQYRGVTFYRRTGRWE
SHIWDCGKQVYLGGFDTAHAAARAYDRAAIKFRGVEADINFNLEDYEGDLKQMTNLTKEEFVHVLRRQSTGFPRGSSKYRGVTLHKC
GRWEARMGQFLGKKYVYLGLFDTEVEAARAYDKAAIKCNGKDAVTNFDPSIYENELNSTESTDSGADHNLDLSLGGSSSKKNNREFG
DNRGQNPSSMQFDVDWRHNGLRPEKQTAPVDMDARRRDNGYNESETLQLLSKTHLHSPVSLKHNNSHQLQRFGQYMRPGESHM
IQMFPPQFGSSNYQIQFPSGSNGGRIGATNVGDLSLSSSNASSQWQSNLPPQIFAAAAASSGFSQQIVRPQNWSSENGFHHFLMRPS

>Rosa_XP_024186592

MLDLNLNFYTPEMETSAASSFNDSSNDSSSSATLNFLDLQNDVAAPPYDDADHHDYFDGRTIQLFPPQPQPPLRSSTTKQWLGLSSG
GAPQRYSAPPPPPPLAEQRIVPLPEKVKKSRRGPRSRSSQYRGVTFYRRTGRWESHIWDNGKQVYLGGFDTAHSAARAYDRAAIKFRG
TEADINFNVSDYDEDIKQMSKFTKEEFVHILRRQSTGFARGSSKYRGVTLHKCGRWEARMGQLLGKKYIYLGLFDSEIEAARAYDQAAI
KCHGREAVTNFNPSLYDGEIMAEANNAGIDDSLDLNLGIAPPLASDCPNENINLSSFPFQLGCNDNPVNMTARNENSVPGLPATMRT
QLPHGSMVASTEPPLMNSMNSNFFPIHMERAMEKSMDANSFPNWAWQMQVLNDGATPVPLFSAAASSGFPSSTTESSAPIPPLHF
PNTSFLQRHFSPSFTNKTPHFYCRS

>Rosa_XP_024157287

MLDLNLSVVGSSSEQNDVFVVGSFPEGSGSATQMDESGTSNSSVVNADASSSNDDSCSARATVTTFNFDILKVGGGEEDDVAVTKEL
FPVNSWPGKGQGQQYSPASSSARHNLIELGGPAEVQQKQPQPQPPPPPPQQPQVKKSRRGPRSRSSQYRGVTFYRRTGRWESHIW
DCGKQVYLGGFDTAHAAARAYDRAAIKFRGVDADINYNLSDYEEDMKQMKNLTKEEFVHILRRQSTGFSRGSSRYRGVTLHKCGRWE
ARMGQFLGKKYIYLGLFDSEVEAARAYDKAAIKCNGREAVTNFEASTYEGEMISDAVNEDGNHNLDLNLGISPPSFGNGQKQGEGHL
QFHSGPYEEQNGKNMRMVPNANATMTASPFRGVIMTSEHPPLWNGVYPSFLPNQERAIEKRIALGSQGPPNWAWQMHGQVSAP
MPLFSTAASSGFSFTATSPSAAVLPIIPSNPTALNLCFTSSATAAHHTSQ

>Rosa_XP_024176749

MLDLNVDVISSTVDQSSYDDKARALEAEKLREDSGSSDSSVVNAEEDSYSNNTSPFIFDILKKDKHGLCNNKEDQIVPSPEFATRSLFPVT
GDGGGGGGELGLLASSNSTSRPQWLNLSFAESGGQAELKVVQQKQQQARKSRRGPRSRSSQYRGVTFYRRTGRWESHIWDCGKQV
YLGGFDTAHSAAKAYDRAAIKFRGVDADINFVLSDYEEDLKQMRHLNKEEFVHVLRRQSNGASRGNSKLRGVAAAAAAAAVPKIGP
FEVRMGQLIPGNKLIKCNGREGVANFDPSIYEGAVVVDASTEGSGHNLDLSLGISQPSTGQKRKAFEINTKEKPMVNGSASAAGGQPL
NATAMLSKHPALYSEIYPGFLHKTEEMATDYHKRIQAVSSPRFSNWAWQMNGNNNNVTPMQVFSIAASSGFSSSIATVPPSATNQL
PPILQDNSTYNLRHPFPTSL

>Peach_Prupe.6G242400

MLDLNLSFVCNDVVSSSDNNLLHLPATTSPIQSSASFNSSSNLTATGDDEDLNFLSPNDVAADDNAHCDARTIQLFPLAQSVRSSSSSSS
SSRKQWLGLSSNSGLEVEPSYYAPAEQIVPLQHKVKKSRRGPRSRSSQYRGVTFYRRTGRWESHIWDCGKQVYLGGFDTAHAAARAY
DRAAIKFRGTEADINFNVSDYEDDIKQMSNFTKEEFVHILRRQSTGFSRGSSKYRGVTLHKCGRWEARMGQFLGKKYIYLGLFDSEIEA
ARAYDKAAIKCNGREAVTNFEPNSYDGQIMSEAHNGGSDKSLDLNLGIAPPLVSELQKNNSNLSSFPVQLGCDDIPIHMRTRNENCAP
APMRAQLSHGSMVASEEPPIMSNINSSFFPIQMERATEKRMDVNSFPNWAWQLQGLNGGATPMPLFSAAASSGFPSSTATSPPAA
VTQLHFPNTTILHHHFSPSTVTNNIPGFYCRS

Figure S1

>Peach_Prupe.6G091100

MLDLNLNVVGSGPNDVESCGTQMDESGTSNSSVVNADASSTNDDSCSTRAARYDAVTTFNFDILKVRGGEDEEDDVVVTKELFPVT
GGLSNWPGQGQSSASSSLVRKNLMELGFDHGGTGEVRLVQQKQQQPAAPPPQQQQVKKSRRGPRSRSSQYRGVTFYRRTGRWES
HIWDCGKQVYLGGFDTAHAAARAYDRAAIKFRGVDADINYNLSDYEEDLKQMKNLTKEEFVHILRRQSTGFSRGSSRYRGVTLHKCGR
WEARMGQFLGKKYIYLGLFDSEVEAARAYDKAAIKCNGREAVTNFEPSTYEGEMISEAGNEDGDHNLDLNLGISPPSFGNCQKEVEG
HLQFHSGPYDGHNGKRMEHNVNATMSDPPFKGLVMTSQHPPLWNGVYPSCFSNQERATEKRIALGSQGPPNWAWQMHGQVSA
TPMPLFSTAASSGFSFSATTPPAAVHPLQPSTPTALNLCFTSPATAAANTSQ

>Peach_Prupe.2G220100

MLDLNVNITLTSDASFDYEKTKDMEVEELPQGSRTQMEDSGTSNSSVVNAEEAPTPSNAGEEDSTNNTTSSFVFDILKKDKDGLCNTT
YYGGAKDQNPSLQFVTRSLFPVTGDGGGGNEAECGLGLSSASSTARPQWLNLSFAESGGQAQAELRIMQQKKPQPRKSRRGPRSRS
SQYRGVTFYRRTGRWESHIWDCGKQVYLGGFDTAHSAARAYDRAAIKFRGVDADINFTLGDYEEDMKQLGHLNKEEFVHVLRRQST
GASRGNSKYRGVALPKCGGAGGRWEARMAQFPEKKVFEKEGIKYNTGREAAAVTNFVDPSIYEGEVVLDASIEGSGHNLDLSLGISQP
SSGQKGNGNLGDFQFRYKERPMVNGSAASAAVGQTPHVLTMVAKHPALYSGMYPGFLQKYEEMDSDHNGAQAVSSPRYTNLAW
QVHGNSHSVSPVQVFSIAASSGFPSSMASTAPPAANYFPPNLQGSASASYNVGPLPFPTPSTM

>Arabidopsis_TOE1

MLDLNLNADSPESTQYGGDSYLDRQTSDNSAGNRVEESGTSTSSVINADGDEDSCSTRAFTLSFDILKVGSSSGGDESPAASASVTKEF
FPVSGDCGHLRDVEGSSSSRNWIDLSFDRIGDGETKLVTPVPTPAPVPAQVKKSRRGPRSRSSQYRGVTFYRRTGRWESHIWDCGKQ
VYLGGFDTAHAAARAYDRAAIKFRGVDADINFTLGDYEEDMKQVQNLSKEEFVHILRRQSTGFSRGSSKYRGVTLHKCGRWEARMG
QFLGKKYIYLGLFDSEVEAARAYDKAAINTNGREAVTNFEMSSYQNEINSESNNSEIDLNLGISLSTGNAPKQNGRLFHFPSNTYETQRG
VSLRIDNEYMGKPVNTPLPYGSSDHRLYWNGACPSYNNPAEGRATEKRSEAEGMMSNWGWQRPGQTSAVRPQPPGPQPPPLFSV
AAASSGFSHFRPQPPNDNATRGYFYPHP

>Arabidopsis_TOE2

MLDLNLDVDSTESTQNERDSITVKGVSLNQMDESVTSNSSVVNAEASSCIDGEDELCSTRTVKFQFEILKGGGEEEEEDDDERSAVMM
TKEFFPVAKGMNFMDSSAQSSRSTVDISFQRGKQGGDFIGSGSGGGDASRVMQPPSQPVKKSRRGPRSKSSQYRGVTFYRRTGRWE
SHIWDCGKQVYLGGFDTAHAAARAYDRAAVKFRGLEADINFVIGDYEEDLKQMANLSKEEVVQVLRRQSSGFSRNNSRYQGVALQKI
GGWGAQMEQLHGNMGCDKAAVQWKGREAASLIEPHASRMIPEAANVKLDLNLGISLSLGDGPKQKDRALRLHHVPNNSVCGRNT
MVQFFLQCTKYDLLYFHMRFAATMENHMAAAACDTPFNFLKRGSDHLNNRHALPSAFFSPMERTPEKGLMLRSHQSFPARTWQG
HDQSSGGTAVAATAPPLFSNAASSGFSLSATRPPSSTAIHHPSQPFVNLNQPGLYVIHPSDYISQHQHNLMNRPQPPP

>Petunia_PhBOB

MFDLNLSCDDVPEESEHLNELSVTQMENSRTSSTTGDDDNSCSDHMSNCYTFDILKTNRKESENLGSFVTKELLPLSLNDQRMVLAQQ
QRLQVIKKSRRGPRSRSSQYRGVTFYRRTGRWESHIWDGGKQVYLGGFDTAHAAARAYDRAAIKFRGLDADINFNVSDYEEDLKQM
KNFSKEEFLHILRRQSTGFSRGSSKFRGVTLHKCGRWEARMGQLLGKKYIYLGLFDSEIEAARAYDKAAIKSNGREAVTNFELSTYEGVLS
SETADIGGTSHNLDLRLGISPSSCADNQHGNTSQMGISQCRPGSNGLPEHREVLSSASTTPRSMLLHGQHMLDQHPLHWNGPNDNL
FPTFKGTSIEKGLEVDTSSKWMRQDQNSYGGSPTAPFFSTAASSGFVNSAVSAPSAVVHQLHFPSRALPYHYSPSLTNETFTLLLQELK

>Petunia_PhBEN

MFDLNLCFEDEEELQFDNHNNSTETSNNSSSIINNIETTTTSSTCDDHEYISYSNNEYNNNSFVDFLKTDNNDQFLDSKELFPLSNGGEM
AAPVNVYGNYGGTMEQRIIIPVQQQQQQQQQQVKKSRRGPRSKSSQYRGVTFYRRTGRWESHIWDCGKQVYLGGFDTAHAAARA
YDRAAIKFRGLDADINFNVSDYQDDLKQMTNFTKEEFVHILRRQSTGFSRGSSQYRGVTLHKCGRWESRMGQFLGKKYIYLGLFDSEIE
AARAYYKAAIKCNGREAVTNFELSTYEGELSTEADNGGASHNLDLNLGIASSSIADDQHDNTCLIGNSEFQCASIGLPEYRGAMNSPCT
TMGSKMPHGRHLLWNGVNTSVFPTFKGTAIGKGMEVDSAPNWTWQDQNLYGGSSSVPLFSTAASSGFANSTTADVHQHYFSTGP
LPYHHSPSLANMNFAQYYCRS

Figure S1

>Petunia_Peaxi162Scf01024g00326

MLDLNVSAVSVNSNCDETDPYNNTNTFLKDDISGTSNTDSSSVVNAVVGDEDSNSSSHQHVFKHSLSTLSFSILKSDRVMETEDDMTS
DDYKTRQLFPVKVETKIQDQAQCWLNLSVPESRGGADIGVYKPPPAKKSRRGPRSRSSQYRGVTFYRRTGRWESHIWDCGKQVYLGG
FDTAHAAARAYDRAAIKFRGVDADINFSITDYEEDMKQMKNLSKEEFVQILRRQSTGFSRGNSKFRGVTLQKCGRWEGRMGQFLGK
KDYDKAAITCNGREAISNFQPNTYGREIKEDNKDGGSGENLDLNLWIAPPWEGPKGDEIGRNVHFKFGAGEMVIGKRLEIESSSTAPQ
GPPTASKCSILTGMYPGFSPHNWEGGMTKAEATSSPGFPNWAWKIRSQGMATPVPVFSSSAASSGFSTTTTLYPNSLLPPSNQLRLPT
NSTFPHPPMTLNIS

>Petunia_Peaxi162Scf00072g00229

MLDLNVSIINNYILDQNLPQVSPSSGTSNSSIQNAEATSSVDDTCSTRAGTSFVTRQLFPIESNREQDLTRSDRVNFTSGFGNVVIVQQQ
QQQEQQQQQAHVKKKSRRGPRSRSSQYRGVTFYRRTGRWESHIWDCGKQVYLGGFDTADVAARAYDRAAIKFRGVDADINFSITD
YEEDMKQLKHLGKEEFVHVLRRQSNGFSRGSSKFRGVTLHKCGRWEARMGQFLGKKYIYLGLFDSEVEAARAYDKAAIKTNGREAVT
NFEPSAYEGETISEPQSEGSHHNLDLNLGISTSSSKENDRFGGKYYHPYDTQDVTKSKMDKPGPVVVGSSYLKGLPMRQPHLWTGAYS
SFSPGYEGRASDKSKDIGSSQGPSNWALQMPGQGGTAPMTMFYTAASSGFVTPATASASPMVPSTIASQYYYQISSHIPPP

>Petunia_Peaxi162Scf00389g00028

MMLDLNLSAIYDEKVGEIAVADESGTSNSSARNAEASSSAGDDDSCSTRAAGDLFAFNFDILKVGGAETSRSFSINNDEEVYDENHMR
MARPDVVTQQFFPVDTAESNRAHHTSRRPDWVDLSYDPPNTLGFREVGIVHPQQQQYQQQQQQQQPVKKSRRGPRSRSSQYRGV
TFYRRTGRWESHIWDCGKQVYLGGFDTAHAAARAYDRAAIKFRGVDADINFNLSDYDEDMKQMKNLSKEEFVHMLRRQSTGFSRG
SSKYRGVTLHKCGRWEARMGQFLGKKYIYLGLFDSEVEAARAYDKAAIKCNGREAVTNFEPSTYEGETISDPRSEGSQQNLDLNLGIST
SSAKEIERSGGFQYHPYDMQDTTKLQMDKSGPAIVSSSHFKAQPVTSEQAHLWNGVYSNFFPSYEERASGKRVEVGSSQGPPSWVM
QMHGQVGTTPMSMFTAAASSGFSSPATIASASPISGPNPNIHNLSFATYSTPSTNTSQYFYQIRPPLPPP

>Ntomentosiformis_XP_018630941

MFDLNLSSEDVPVESGTSNSSIVNMETSNSTAGDDEYEESGNSDFVLKELFPLVSGEAELSQQQCLDLSANCGASNEQRIVISPQRRSQ
VKKNRRGPRSRSSQYRGVTFYRRTGRWESHIWDGGKQVYLGGFDTAHTAARAYDRAAIKFRGLDADINFNISDYEDDLKQMNNFSN
EEFVHILRRQSNGFSRGSSKYRGVTLHKCGRWEARMGQLLGKKYIYLGLFDSEIEAARAYDKAAIQSSGREAVTNFEISTYEGVLSSEADI
RGTGHNLDLNLGISPSSYDDNQHGNTSQTGNFQGQHGSNGLTEHHRKVLNSASTTLRSELLLQGQHVLTQHPLHWNGLNGNLFPTF
KGTSIEKGLEVDTSLKWTQQGQNTYGGRPTV

>Ntomentosiformis_XP_009606014

MFDLNLGFEEFAMDDDTVHLSYETTTSEKLQELQMENSGTSNSSIVNIETSSTAGDDEFFSCSDDRRRNDGYAFDILKGNDYEESEFVT
KELFPLTGGEAATELLSRQQWLDLSANYGGPAEQRIVVIPQQRQQVKKSRRGPRSKSSQYRGVTFYRRTGRWESHIWDCGKQVYLGG
FDTAHAAARAYDRAAIKFRGLDADINFNVADYHDDLKQMSNFTKEEFVHILRRQSTGFSRGSSKYRGVTLHKCGRWEARMGQFLGKK
YIYLGLFDSEIEAARAYDKAAIKCSGREAVTNFELSTYEGELSAEDNGGASHNLDLNLGIASSSQADEQHGNTCQMGNTEFLLASNGLTE
YRGAMRSPSTTMGSKLPHGRQPLTDRPLLWNGVNTSVFPTFKGTAIGKGMEVDSSPNWTRQDQHPYGGSPSMPLFSTAASSGFAN
STSAAAHQHHFSTGPLPYHHSPSLANMNFAHYYCRS

>Ntomentosiformis_XP_009611308

MLDLNVNVIYDENYIVDEKLPEGSVADHESGTSNSSSLRNAEATSSAGGDDDTCSTRAGNLFAFNFDILKFGGVDTTSRSNKFVTRQLF
PVHDGELNRPGQTDRVNFSSGRSGFGDERTVLQQQQQSQQEKPQVKKSRRGPRSRSSQYRGVTFYRRTGRWESHIWDCGKQVYLG
GFDTAHTAARAYDRAAIKFRGVDADINFSISDYEEDMKQMKDLGKEEFVHLLRRHSNGFSRGSSKFRGVTLHKCGRWEARMGQFLG
KKYMYLGLFDSEVEAARAYDKAAIKTNGREAVTNFEPSAYEGETISEPQGRQHDLDLNLGMSTSSSKENDKLGGSYYHPYDMQDATKS
KMDKPGPVIVGSSHLKGLPMTSKQAHLWSGASSNFSPTYEGRASDKRKDIGSSQGPSNWALQMPSQVDTAPVSMFYTAASSGFLTP
ATAFITSSASPVAPSTNASQYYYQFSPHVPPP

Figure S1

>Ntomentosiformis_XP_009623868

MMLDLNLSAIYDEKVLEGSVVDESGTSNSSARNAEASSSAGDDDTCSTRAGDMFAFNFDFLKVGGAETSRSCSNNDDDQDGYDENR
RVTQSEFVTRQLFPLDRSHTSRKPDWVDPSFDPPNTVSFREAEIVRAQQQYQQHQQQQQQVKKSRRGPRSRSSQYRGVTFYRRTGR
WESHIWDCGKQVYLGGFDTAHAAARAYDRAAIKFRGVDADINFNLSDYEEDMKQMKSLGKEEFVHVLRRQSTGFSRGSSKYRGVTL
HKCGRWEARMGQFLGKKYIYLGLFDSEVEAARAYDKAAIKCNGREAVTNFEPSTYEGETHSDPQSAGSQQDLDLNLGIATSSPKENER
MGSFQYHPYDMQDATKSQMDKSCPAIVGSSHFKAQPVTSEQAHVWNGVYSNFFPSYEERASGKRIDIGSSQGRPNWALQMHGQV
GTTPMSMFTAAASSGFSTPATTASASPMSGPNPNTSTPNLPFASYSTPSTNTTQYYYQIRPPLPPP

>Ntomentosiformis_XP_009600856

MLDLNVSAVSVNSNCDETDPNTNTLFFKDEYSAGTSNTTASSSVVNAPVAGDEDSNSSSVHHGSKRAHHSLSTLNFSILKSDRAIEIED
DMSTDDVLTRQLFPVKNESQGQGTRIQGQSQCWLNLSVPESGGREETGIYKPPAPAPAQQPVKKSRRGPRSRSSQYRGVTFYRRTGR
WESHIWDCGKQVYLGGFDTAHAAARAYDRAAIKFRGVDADINFNIIDYEEDMEQMKNLSKEEFVQVLRRQSTGFSRGNSKYRGVTL
HKYGRWEARMGQFLGKKAYDKAAIKCNGREAVTNFEPNTYEQDIKEDNKDGGSGQNLDLNLWISPPLAGPKRNEIGRNVHFNFGAR
EMVIGRRFEIANSSTAPLGPPTASKLSLLTGVYPGFSPHNMEGAMMKVEATSSPGFPNWAWKIHSQGVVTPVPVFSSSAASSGFSTA
TTMYPNTLIPPGNQLRLPTNSTFQHPPVTLNIS

>Vitis_XP_002273339

MLDLNLNDVSDDSFHGSTQAVDPGKLLDLSSNQMEDSGSFNSSIVNADALSSTTADDDSSSNHLDHCCGIVAFSFDFFKQNEDHDNK
SSDSVTRQFFPVTSGGEGDCSLGSSLQHPCILQVGSGGPLLPKKPQQQQQVKKSRRGPRSRSSQYRGVTFYRRTGRWESHIWDCGKQ
VYLGGFDTAHAAARAYDRAAIKFRGADADINFAVSDYDEDIKQMSNFTKEEFVHVLRRGSTGFSRGSSKYRGVTLHKCGRWEARMG
QFLGKKYIYLGLFDSEIEAARAYDKAAIKCNGREAVTNFEPSTYEGEIIFPADNGGSSQNLNLLPNLDLHLGVAPYFSDEPMSDEPVRNSK
ANGSPSRQGHDAMPDDRGERIENSTSATLGVHPSYGLVMASDHPPSWSGVNSRVFPIYKERAIEKRIEVDHLPNWAWQNHGPYGG
ATPPLFSTAASSGFLSSVTTASPANPTQGSLQSTTVLQHYYSPSLTNTTSISPYYYCRS

>Vitis_XP_002275627

MWDLNVESGCDEDERMIVVVVEKGEEEELVPGGCRSLMEDSGTSNSSVVNADEAPSNGGDEDSSNNSSAFNFGILNKLGRHVPTYG
AVEETPEFVTRQLFPATGDRGGGESELCSGSSSTSFPKPQWLNLSCPEPIGQQKPKQQQQQQQQVRKSRRGPRSRSSQYRGVTFYR
RTGRWESHIWDCGKQVYLGGFDTAHAAARAYDRAAIKFRGVDADINFSISDYEEDMKQMKNLNKEEFVHILRRQSNGFSRGSSKYR
GVTLHKCGRWEARMGQFLGKKYIYLGLFDSEIEAARAYDKAAIRYNGREAVTNFVPSTYGEGAILEANDRSSGHNLDLNLGLSFPPDGP
RGNDTVGRRSMVEGSTYTLVGGQPRHSLTTVSKHLPMISGICTSFLPIHEETALEKRLDGVSSPRFSNWARQVYGHGVNIPIPLLSTAAS
SGFPSCSTIGPSATLPLLNPRNINPALQPPPQISPPTTSTTNSTSCFYNFGSSNQQMQF

>Vitis_XP_002284749

MLDLNLNVGSSDSTQHGDSVVGSEKFPEGSGTQMDESGTSNSSIVNAEASSNGGGDDDSCSTRAGDAFSLNFDILKVGDCGSPNDV
VTRQLFPMSAGGGVGGDSVFFQGQSSSNWIDLSFNQSGTVGGQEVRVAQQHPQPQQQQQPVKKSRRGPRSRSSQYRGVTFYRRT
GRWESHIWDCGKQVYLGGFDTAHAAARAYDRAAIKFRGVDADINFNLSDYDEDLKQMKNLTKEEFVHILRRQSTGFSRGSSKYRGVT
LHKCGRWEARMGQFLGKKYIYLGLFDSEVEAARAYDKAAIKCNGREAVTNFEPSTYEGEMIPEASNEGSDHNLDLNLGISPPLSNGPKE
IDGLGHLQFHSGSHDAHNGRRSMMENPNIETAGDPSFKGLAMTSEHPPLWTGVYPSFFPGYEERATERRIEASSSQGLPNWGWQM
HGQVTATPMPLFSTAASSGFSSSATTAPSAAILSSKPPNTTSHSICFASSASAVTHPSQYYFQMKPPPP

>Dianthus_Dca21030

MVDLNLTLHPPDSTSGYDTVSNAPTFNFEGILGTSNCGYLGNDRGVYGPSRVGYVVMGQGLSQVKKSRRGPRSRSSQYRGVTFYRRT
GRWESHIWDNGKQVYLGGFDTAHAAARAYDRAAIKFRGVDADINFDVSDYEDDLKQMSNLTKEEFVHLLRRQSTGFSRGSSKYRGV
TLHKCGRWEARMGQLLGKKYIYLGLFDSELEAARAYDKAAIQCNGKEAVTNFDPTTYGDELMDETPFGGGPNSLDLNLGIGNASSAD
GHVKTHNLDGMLNMRAKSQNRISAVEMPPICHGHRYLSELPTTWSDINSMSLPVYQERAVEKVAESDPWTTWARKLQGTQTVPFY
ANAASSGFPSIATASTAAFGQQQYSNSTSLHYSYPL

Figure S1

>Dianthus_Dca22443

MMFDLNNSAHNFEKTTTETTIKEDSATNTELSGTSSSSSVVNADDSPLSSAADPSFVTRQLFPLTAADNRLAGGGGETSEMEVLRLGTT
PNQLSNNNQWLNLGVSGSGSGSVEVGNNKQQQVRRSRRGPRSRSSQYRGVTFYRRTGRWESHIWDCGKQVYLGGFDTAHSAARA
YDRAAIKFRGVDADINFNITDYDDDMKQMKNLSKEEFVQVLRRQSNGFSRGTSRYRGVTLHNKCGQWESRVGQFLGKTPYDQAAM
QRNGTEAVANFDPSTYENPIPLQAHHIGDGHNLDLNLGISITIDGPKTSNNMGVRESQTHQSPTLLADGKRPMNGASTCYIPGGAHPL
YGQPTASHTLYEVMYRTGHLLAIYLVVHILYMDSQRHRDNLVLCGWDCHKVSLQHMRKRRMTRKPKPFRLLQGCRTGHGTCKTKAT
VKVKVKVEAAVQAKSNNRQLQHHQDSRSLPPHRHQRAPRFTITVKEKANDQKAEAISSAARLSNWAWYMQNQGHGQGQGQGGG
GSSSQVQQSSTAASSGFPFSTATPSSTSTTFHHYGQGRS

>Spinacia_XP_021838734

MLDLNLTVFSHDYSNSVQNNNSFTSNNSSSLDNGDSLSSNNSPSTITYNFDILRLDTSLNNDGDCCNYYPVDRGEASSTGFVTQDLFPA
CDGPGRPIVWGQNPLMGVLEVRSMVMGQAQAQAQAQAQKSQQVKKKKSRRGPRSRSSQYRGVTFYRRTGRWESHIWDNGKQV
YLGGFDTAHAAARAYDRAAIKFRGVDADINFDVSDYEDDLKQMHSLTKEEFVHLLRRQSTGFSRGSSKYRGVTLHKCGRWEARMGQL
LGKKYIYLGLYDSEVEAARAYDKAALQCNGKEAVTNFDPSIYGEELISEPDVGGGHYTLDLNLGIGNPSFADNDVSYPLLHEQNCRLSD
MREKDQNWIQPATGPTAHDGHSFPSQQPSAWCGVNPIFFPVYQEKAMEKTREIDPWSNWSWQIQHQGAHGGSMPVPVFSNAA
SSGFPSITTASSAAVGRTHYSN

>Spinacia_XP_021865446

MNNRMFDLNMMDVVANNNNSMNNKEERENSMNDEGSATNTELSGTSSASSLVNATAEESPISGAGEFDFFSKHSSADVSPFVTR
QLFPMNEGDNGGGSVGYDGGGDGTREMELLRLGSSSSTPRSMPTNHNSNSNQWLNLSVLGSGPGSGVGTEQQNNAKQQVRRSR
RGPRSRSSQYRGVTFYRRTGRWESHIWDCGKQVYLGGFDTAHAAARAYDRAAIKFRGVDADINFTIADYEDDMKQQMKNLSKEEFV
HILRRQSTGFSRGSSRYRGVTLHKCGRWEARMGQFLGKKAYDKAAIQCNGREAVTNFESSSYQGQIPIMQPNNQGDGQNLDLNLGI
SITIDKGAGESQPHHPPLHLPNGNWPANGSYFIPGAPQLYGQPLASRQSTPMWSGLPPGRPATYEERVSDQQKLEAIAGSARLPNW
GWYMQNQGGQGGGSGGTANQVQQLSNAASSGFSSFSTTTTSSANYPFTTLHNDFHNNNNNNNNNHTQGR

>Camellia_XP_028106735

MLDLNVDSSCNEVEATKITKLPRSDDCSGTSISSIVNNNTPDEDSTSNPPSSSVCSFNFDIFSSKAKDPSIIIAIQDDSDENSPPQFVTRQFF
PSGHSDEFDSRSSSLRPPQCVNLSFSDSGGVPELTNFQQQQHKQPQQQQVKKSRRGPRSRSSQYRGVTFYRRTGRWESHIWDCGKQ
VYLGGFDTAHAAARAYDRAAIKFRGLDADINFSLSNYEEDMKQMTNLTKEEFVQILRRRSTGFSRGSSKYRGVTLHKCGRWEARMGQ
FLGKKYIYLGLFNSEVEAARAYDKAAIKCNGREAITNFEASTYEGEISFDTNIPDGSTAQNLDLNLWISAPTDVPKGNDNVRNFQSQYAA
CEMPDGKRPKVETSVSSPIDGRNIQGLTMASKNSSVWSGNHPSFTPNYEERALGKVTEAIPSPAFPNWAWKMQNQSVDSPVALFSA
AASSGFPPSTPTTTLSATLAPQNLQNITPYFPTQTTNISHFYNHKS

>Camellia_XP_028125723

MLDLNVDVVTEESTCDELEKKKIATRKLLEGDDSVTSNSSVVVNADEAPNNAADEDSNSNMISPFNFDVFSRGKGFGVGNIVAIGDEC
DRNLAPLLVTRQLFPVGDSEEIGSGSSSSATSLLRPQWLNLSFPDSGAAAGLKAEKKPQPQQQPVKKSRRGPRSRSSQYRGVTFYRRTG
RWESHIWDCGKQVYLGGFDTAHAAARAYDRAAIKFRGVEADINFTIKDYEDDMKEMTNLTKEEFVHILRRQSTGFSRGSSKYRGVTLH
KCGRWEARMGQFLGKKYMYLGLFDSEVEAARAYDKAAIKCNGREAVTNFEPSTYEGEMISTDANNADGGTSQNLDLNLWIFPPADV
PKGNVNVRDIQSHCAACEMPDGKRPKVESSISTPTDGQNTRGLTMASKHPPIRSGIYPSFTPNYEERAIGKSTEALPSLAFSNWAWQ
MHSHSVVSPVAPFSTAASSGFSSPNSTAALPATFPPQNKTAHKLYIPTPTTNISHFYNH

Figure S1

>Camellia_XP_028061878

MLDLNLSVVHNENSMAITEKFPDGSGGQMDESGTSNSSIVNVEVSSNAGDDDDSCSTRVPGRDIFAFNFGIMKLGGECNDNTTEEG
KEDRMTQSEFVTRQLFPVSGDPGLTVGQSLAPSPGALLRPNLVDLSFGQASSGGLQEVRVLQPQQQQVKKSRRGPRSRSSQYRGVTF
YRRTGRWESHIWDCGKQVYLGGFDTAHAAARAYDRAAIKFRGVDADINFNLSDYEEDLTQTKNLTKEEFVHILRRQSTGFSRGSSKYR
GVTLHKCGRWEARMGQFLGKKYIYLGLFDSEIEAARAYDKAAIQYNGREAVTNFEPSTYDGETISEVNGGGGDHNLDLNLGISTPSSG
DGPKDNKNSGHLQFHPYGIQDARRSNMKNPSTTTIGNPTLEGLAMTSEHCPLWTGGVYPNFFPSYEERATEKRIEVGSSQGPPNWV
WQMHGRVTPTQMATFSTAASSGFSSVSTTALSASIHPSNPPNPTALNLCFSSSATASGNPQYYYQMRPLQPPP

>Camellia_XP_028098446

MLDLNLSILDNEVLANVPESSEEESGTSNSSVVNAEASSSNAGDDDSCSTRGDIFAFNFDILKVTAATTESEVEGNENRMTRSEFVTRQL
FPSIGGVGGLTQGLSSSSPSFLRPNWVDPSLNQATSHGGLAEVRVVKQQVKKSRRGPRPRSSQYRGVTFYRRTGRWESHIWDCGKQ
VYLGGFDTAHAAARAYDRAAIKFRGIEADINFNLSDYEDDLKQMTNMTKEEFVHILRRQSTGFSRGSSKYRGVTLHKCGRWEARMGQ
FLGKKYIYLGLFDNEVEAARAYDQAAIKCNGKEAVTNFEPSTYDGEMISEAYHEGGNHNLDLNLMISAPSSGDGPKDNGSLRHLQFHP
YEVHGARRSNMESPATTTLGNPTLKGQAMTSDHHPLWTGLHPNFFPNYEEKATRRGIEEGSSQGSPNWAWQMHGGQGNTAPTR
VSTAASSGFSSAATTTAAFSASVHPSKLLKPTALHLCFPSSATASSNYQYYYQMRPLQPPP

>Camellia_XP_028097115

MMLDLNLTAVATDSISDGLEAVTEKFLELSGVHMDDSGTSNSSIINLDTSSTNLGDEEFSSSHLYSFDILKTTECEEYNNGVPQTKQLFPV
NGEGGEDASSCQQRQWLDLSVVAGNSGGPKEERGLVVPQQRQQVRKSRRGPRSRSSQYRGVTFYRRTGRWESHIWDCGKQVYLG
GFDTAHAAARAYDRAAIKFRGTDADINFNISDYNEDFQQMKNLTKEEFVHVLRRQSTGFSRGTSKYRGVTLHKCGRWEARMGQFLG
KKYIYLGLFDSEVEAARAYDKAAIKCNGREAVTNFEASTYEEELSSEIENGGSSHNLDLNLGISTPYFANGQMGSNDLSSGLHLQSDLSN
MHENRRAENSVTTMGIQPPHGQIMVSEHPPLWKGVNSHAVPIYEGRAIENSMEFDSSPSWALQFQGPFGGDVPLPLFSTAASSGFA
TSTIATSSAAVYQPWFPTTTIPHNHYFPSVIDSNNISQYYCRS

>Papaya_XP_021891498
MLDLNLGTASSSDSVDVFSDKLLQLSPATQMESSGSFNSSIVNAEATGDDDSCSNTHPLVYSFDILKTNDEYQDSTTRNKKTVRVHAHE
ADDTHDRTIQFFPVSGIGASGTSSLSSNTRQLLDLGCRVVEYGIPLEQGIATQQQPAKKSRRGPRSRSSQYRGVTFYRRTGRWESHIWD
CGKQVYLGGFDTAHAAARAYDKAAIKFRGVDADINFNVSDYTEDIKQMHNFTKEEFVHILRRQSTGFSRGSSKYRGVTLHKCGRWEA
RMGQFPGKKYVYLGLFDSEIDAARAYDKAAIKCNGKEAVTNFEPSSYDGEIILEAENEDGNQTLDLNLGIASSTAQKESNDMNDFCFQS
SRNSMHNDRRARIENSVIVSGKLQPPHGPATVSDHPPNWGGPNSFFPNNKVISERAMEKRMVPNWTWQLETRCLGSNPVPLFSAA
ASSGFLSSKSITPPTAANQVYHPNATTMTVYRKHPVNLPANTTNPNVSFYYCSS

>Papaya_XP_021910160

MLDLNLDVLVASESSSCDNHREDEKTMTVLAEPYKLTAEARAGSVRTQMEEEYSGTSNSSIINSLDEAPTNAGDEDYSNNTGSAFILTSI
KRGVDDDDDRDEKYPSPDSVTQQLFPVAGEKSGPGVEFGFGVGSSVRLDRQWLNLSCAKGSAEAIEGQKIVPQKVKKSRRGPRSRSS
QYRGVTFYRRTGRWESHIWDCGKQVYLGGFDTAHAAARAYDRAAIKFRGDDADINFSLSDYKDEIKQMRHLSKEEFVHILRRQSTGFS
RGSSRFRGVTLHKCGRWEARIGQFLGKNAYDMAATKCNGREAVTNFEQSNFKINGGSGQNLDLSLGISLTPSRRENVGDLQACSSET
PLKGMSLLETSASAYLGGQQPNPNTLLSGMYPVSSPNLREQTGERAIEAASSMRSSLSWTWQSNGNVGQMPILNIAASSGFSSTATL
QQKIHYNNNNPTQNLCLVNHQKFS

Figure S1

B

>Bnapus_XP_013714881.1

MLDLNLDVDSKEVSGAVLNQMDESVTSNSSLVNAEASSCIDGEEELCSTPAVKFQFEILKGGGREDEEGRSEVMTKEFFPIEKGASFGD
SSGQSSRSSVDISFQRGNQGRDAARVMQPPAQPVKKSRRGPRSKSSQYRGVTFYRRTGRWESHIWDCGKQVYLGGFDTAHAAARA
YDRAAVKFRGLEADINFVISDYEEDLKQMANLAKEEVVQVLRRQSSGFSRNNSRYQGANLPKIGGWGGAQMEQLNGNMAYDKAIA
TKWNGREAASLIEPHAPRIIPAAANAKLDLNLGISLSLGDSPKQNLNNVVCGRNSKMENHMAASTCDTPFNFLKRGSDHLINRHVHPS
AFFSPMERTPKEGFMPRSPQSFPARTWQPQDQPSGGTATTATATPLLSNAASSGFSHSATHPPSSSTATLHPSLPFLNLNMPGLYVIH
PSEYASHHHHDINRSQPPP

>Bnapus_XP_013685425.1

MLDLNLDLDSKEVSGAVLNQMDESVTSNSSLVNAEASSCIDGEEELCSAPAVKFRFEILKGGGREGEEGRSEVMTKEFFPVEKGASFVD
LSGQSSRSSVDISFQRGNQGGDAARVMQPPAQPVKKSRRGPRSKSSQYRGVTFYRRTGRWESHIWDCGKQVYLGGFDTAHAAARA
YDRAAVKFRGLEADINFVISDYEEDLKQMANLSKEEVVQVLRRQSSGFSRNNSRYQGANLPKIGGWGGAQMEQLNGNMAYDKATA
TKWNGREAASLIEPHASRIISETANVKLDLNLGISLSLGDSPKQNLNNIVCGRNSKMENHMAASTCDTPFNFLKRGSDHLINRHVHPSA
LFSPMERTPREGFMPGSPQSFPVRTWQAQDQPSGGTATTATASTLLSNAASSGFSHSATRPPSSSTASLHPSQPFLNLNIPGLYVIHPS
QHQHHHDINRSQPPP

>Bnapus_XP_013681156.1

MMLDLNLDVVSAESTHMDESVTSNSSVVNAEASSCIDGEEELCSTRAAVKFQFEILKGRGGEEEEEDRKTKEFFPVAQSSRSSVDISFQ
RGTQGGDFVQPPPAQPVKKSRRGPRSKSSQYRGVTFYRRTGRWESHIWDCGKQVYLGGFDTAHAAARAYDRAAVKFRGLEADINFII
SDYEEDLKQMANLSKEEVVQVLRRQSSGFSRSNSRYQGVSLQKIGGWGAQMKQFNGNMAVASLIEPHASRIIPESANVKLDLSLGISL
SLGDGPKQKDKAPRRHHPPNSMMDNHMTAATCDTPFNLLKRGSDHLLNRHVPHPSAFFSPMERTPEKGFMSPSPQSFLARTWQA
QDQFSGGTATTAIASPLYSNAASSGFSPSATRPPPSSTASATLHPSQPFLNLNMPGLYVIHPSDYSSQQHHHHLMNRSQPPPP

>Bnapus_XP_022551601.1

MMLDLNLDVVSTQMDESVTSNSSVVNAEASSCIDGEEELCSTRAAVKFQFEILKGRGREEEDRTTKEFFPVAQSSRSSVDISFQRGTQG
GDFVQPPPAQPVKKSRRGPRSKSSQYRGVTFYRRTGRWESHIWDCGKQVYLGGFDTAHAAARAYDRAAVRFRGLEADINFVISDYEE
DLKQMANLSKEEVVQVLRRQSSGFSRSNSRYQGVSLQKIGGWRGTQMKQFNGNIDYSDGSYDYGIRTVASLIEPHASRIIPEAANVKL
DLSLGISLSLGDGPKQKDKTPNSMMENHMAAATCDSPFNLLKKGSDHLINRHVLHPSAFFSPMERTPEKGFMSPPPQSFPTRTLQAQ
DQFSGATATTAIASPLYSNAASSGFSPSATPPPSSSSTATLHPSQPFLNLNMPGLYVIHPSDYTSQQHHHHLMNRSQPPP

>Bnapus_XP_013733814.1

MLLDLNLDADSPESTQYGDSNADRQTSDGSGNRVDESGTSTSSVINADADDDSCSTRAFTHSFDILKVGGGDESTASIAGVTKEFFPV
AGDCGHLRGSSSRSWMDLSFDTKSVAPAPAPAQVRKSRRGPRSRSSQYRGVTFYRRTGRWESHIWDCGKQVYLGGFDTAHAAARA
YDRAAIKFRGVDADINFTVGDYEEDMKQVQNLSKEEFVHILRRQSTGFSRGSSKYRGVTLHKCGGWEAGMAQLLAKKAYNKAAISSN
GREAVTNFELSTYQNEINSESGHDKLDLNLGISLSPGNAAKQNGRFSHFPSNPYETPPGVSWTIDTEFMGKPVNTPLPYGSSDHRANF
KENISEAEGGMMSNWGWHRPGKTSTMRPQQPGAQPPQLFSVAAASSGFSNFRQQPPSENASHGYFYPQF

>Bnapus_XP_013734915.1

MMLDLNLDADSPESTQYGDSNADRQTSDGSGNRVDESGTSTSSVINADADDDSCSARAFTHSFDILKVGGGGGDESTASISGVTKEL
FPVAGDCGKLRGWSSRSWMDLSVGTKSVAPAPAPAQVRKSRRGPRSRSSQYRGVTFYRRTGRWESHIWDCGKQVYLGGFDTAHAA
ARAYDRAAIKFRGVDADINFTVGDYEEDMKQVQNLSKEEFVHILRRQSTGFSRGSSKYRGVTLHKCGKWEAGMGQLLAKKAYNKAAI
SSNGREAVANFEHSTYQKEINSESGHDKIDLNLGISLSPGNAVKQNGRLSHFPSNPYETPPGVSLTIDTEFMGKPVNTHLPYGSSDHRA
NFEENISEAEGGMMSNWGWHRPGQTSTMRPQQPGAQPPQLFSVAAASSGFSNFRQQPPSENASHGYFYPQF

Figure S1

>Bnapus_XP_013653876.1

MLDLNLDADSPESTQYGDSYADRKTSDGSGNRSETSTSSVINAEADDDSCSTRAFTLNFDILKVGSSGDGCGGGGDESTAVTKEFFPV
AGDCGHLRGSSSRSWIDLSFDGSRQGETNLVAPAPAPAPVKKSRRGPRSRSSQYRGVTFYRRTGRWESHIWDCGKQVYLGGFDTAH
AAARAYDRAAIKFRGVDADINFTLGDYEEDMKQVQNLSKEEFVHILRRQSTGFSRGSSKYRGVTLHKCGRWEARMGQFLGKKYVYLG
LFDSEVEAARAYDKAAISSNGREAVTNFELSSYQNETNSETNNQGAHDKLDLSLGMSLSPGNASKQNGRLFHYPSNTYETQRGVSLTI
GNEFMGKPVNAPLPYGSSDHRAYWNGYNNPVEGRATEKRSEAEGGGMMSNWGWQGPGQTSAMRPQPPGPQPQLFSVAAASS
GFSNFRQQPPNDNASSLGYFYPQP

Figure S1

C

>Tobacco_XP_016502850

MFDLNLSSEDVPVESGTSNSSIVNMETSNSTAGDDEYEESGNSDFVLKELFPLVSGEAELSQQQCLDLSANCGASNEQRIVISPQRRSQ
VKKNRRGPRSRSSQYRGVTFYRRTGRWESHIWDGGKQVYLGGFDTAHTAARAYDRAAIKFRGLDADINFNISDYEDDLKQMNNFSN
EEFVHILRRQSNGFSRGSSKYRGVTLHKCGRWEARMGQLLGKKYIYLGLFDSEIEAARAYDKAAIQSSGREAVTNFEISTYEGVLSSEADI
RGTGHNLDLNLGISPSSYDDNQHGNTSQTGNFQGQHGSNGLTEHHRKVLNSASTTLRSELLLQGQHVLTQHPLHWNGLNGNLFPTF
KGTSIEKGLEVDTSLKWTQQGQNTYGGRPTV

>Tobacco_XP_016482517

MFDLNLGFEEFAMDDDTVHLSYETTTSEKLQELQMENSGTSNSSIVNIETSSTAGDDEFFSCSDDRRRNDGYAFDILKGNDYEESEFVT
KELFPLTGGEAATELLSRQQWLDLSANYGGPAEQRIVVIPQQRQQVKKSRRGPRSKSSQYRGVTFYRRTGRWESHIWDCGKQVYLGG
FDTAHAAARAYDRAAIKFRGLDADINFNVADYHDDLKQMSNFTKEEFVHILRRQSTGFSRGSSKYRGVTLHKCGRWEARMGQFLGKK
YIYLGLFDSEIEAARAYDKAVIKCSGREAVTNFELSTYEGELSAEDNGGASHNLDLNLGIASSSQADEQHGNTCQMGNTEFLLASNGLTE
YRGAMRSPSTTMGSKLPHGRQPLTDRPLLWNGVNTSVFPTFKGTAIGKGMEVDSSPNWTRQDQHPYGGSPSMPLFSTAASSGFAN
STSAAAHQHHFSTGPLPYHHSPSLANMNFAHYYCRS

>Tobacco_XP_016495732

MMLDLNLSAIYDEKVLEGSVVDESGTSNSSARNAEASSSAGDDDTCSTRAGDMFAFNFDFLKVGGAETSRSCSNNDDDQDGYDENR
RVTQSEFVTRQLFPLDRSHTSRKPDWVDPSFDPPNTVSFREAEIVRAQQQYQQHQQQQQVKKSRRGPRSRSSQYRGVTFYGRTGR
WESHIWDCGKQVYLGGFDTAHAAARAYDRAAIKFRGVDADINFNLSDYEEDMKQMKSLGKEEFVHVLRRQSTGFSRGSSKYRGVTL
HKCGRWEARMGQFLGKKYIYLGLFDSEVEAARAYDKAAIKCNGREAVTNFEPSTYEGETHSDPQSAGSQQDLDLNLGIATSSPKENER
MGSFQYHPYDMQDATKSQMDKSCPAIVGSSHFKAQPVTSEQAHVWNGVYSNFFPSYEERASGKRIDIGSSQGRPNWALQMHGQV
GTTPMSMFTAAASSGFSTPATTASASPMSGPNPNTSTPNLPFASYSTPSTNTTQYYYQIRPPLPPP

>Tobacco_XP_016432764

MLDLNVNVIYDENYIVDEKLPEGSVADHESGTSNSSSLRNAEATSSAGGDDDTCSTRAGNLFAFNFDILKFGGVDTTSRSNKFVTRQLF
PVHDGELNRPGQTDRVNFSSGRSGFGDERTVLQQQQQPQQEKPQVKKSRRGPRSRSSQYRGVTFYRRTGRWESHIWDCGKQVYLG
GFDTAHTAARAYDRAAIKFRGVDADINFSISDYEEDMKQMKDLGKEEFVHLLRRHSNGFSRGSSKFRGVTLHKCGRWEARMGQFLG
KKYMYLGLFDSEVEAARAYDKAAIKTNGREAVTNFEPSAYEGETISEPQGRQHDLDLNLGMSTSSSKENDKLGGSYYHPYDMQDATKS
KMDKPGPVIVGSSHLKGLPMTSKQAHLWSGASSNFSPTYEGRASDKRKDIGSSQGPSNWALQMPSQVDTAPVSMFYTAASSGFLTP
ATAFITSSASPVAPSTNASQYYYQFSPHVPPP

>Tobacco_XP_016454719

MLDLNVSAVSVNSNCDETDPNNNTLFFKDEYSAGTSNTTASSSVVNAPVAGDEDSNSSSVHHGSKRAHHSLSTLNFSILKSDRAIEIED
DMSTDDVLTRQLFPVKNESQEQGTRIQGQSQCWLNLSVPESGGGAEMGIYKPPAPAQQPVKKSRRGPRSRSSQYRGVTFYRRTGR
WESHIWDCGKQVYLGGFDTAHAAARAYDRAAIKFRGVDADINFNITDYEEDMEQMKNLSKEEFVQVLRRQSTGFSRGNSKYRGVTL
HKYGRWEARMGQYLGKKAYDKAAIKCNGREAVTNFEPKSYERDIKEDNKDGGSGQNLDLNLWISPPLAGPKRDEIGRNVHFNFGAR
EMVIGRRFEIENSSTAPQGPPTASKLSLLTGVYPGFSPHNMEGAMMKVEATSSPGFPNWAWKIHSQGVVTPVPVFSSSAASSGFSTA
TTMYPNTLIPPSNQLRLPTKPTFQHPPVTLNIS

Figure S1

>Tobacco_XP_016499635

MFDLNLGFEEFAMDDDTVHVSYDTATSEKLRELQLENSGTSNSSIVNVETSSTAGDDDFFSCSDDRRRNDGFAFDILRGNDYEESEFVT
KELFPLTGGESAAAELLSPQQWLDLSANYGGPAEQRIVVVPQQRQQVKKSRRGPRSKSSQYRGVTFYRRTGRWESHIWDCGKQVYL
GGFDTAHAAARAYDRAAIKFRGLDADINFNVADYHDDLKQMSNFTKEEFVHILRRQSTGFSRGSSKYRGVTLHKCGRWEARMGQFL
GKKYIYLGLFDSEIEAARAYDKAAIKCSGKEAVTNFELSTYEGELSTQDDGGASHNLDLNLGIASSSLADEQHGNTCQMHSPSTTMGSK
LPHRRQPLTDRPLLWNGVNTSVFPTFKGTAIGKGMEVDSSPNWARQDQHPYGGSPSVPLFSTAASSGFANSTSAAAHQHHFSTGSL
PYHHSPSLANMNFAHYYCRS

>Tobacco_XP_016460979

MLDLNVNVIYDENYIVDEKLPEGSVADHESGTSNSSSLRNAEATSSAGGDDDTCSTRADNLFAFNFDILKFGGVDATSRSNAFVTRQLF
PVHDGELNRPGQTDRVNFSSGRSGFGDERTVLQQQPQQEKPQVKKSRRGPRSRSSQYRGVTFYRRTGRWESHIWDCGKQVYLGGF
DTAHTAARAYDRAAIKFRGVDADINFSISDYEEDMKQMKDLGKEEFVHLLRRHSNGFSRGSSKFRGVTLHKCGRWEARMGQFLGKK
YIYLGLFDSEVEAARAYDKAAIKTNGREAVTNFEPSAYQGETISEPQKEGSQPDLDLNLGMSTSSSKENDKLGGSYYHPYDMQDATKSK
MDKPGPVIVGSSHLKGLPMTSKQAHLWSGAYSNFSPTYEGRASDKRKDTGSSLGPSNWAMQMPTQVDTAPVSMFYTAASSGFLTP
ASAFITSSASPVAPSTNASQYYYQFSPHVPPP

>Tobacco_XP_016501408

MMLDLNLSAIYDEKVPEGSVADESGTSNSSARNAEASSSAGDDDTCSTRAGDMFAFNFDILKVGGAETSRSCSNNDDDQEGYDENR
RVTQSEFVTRQLFPLDGSHTSRKPDWVDLSFDPPNTVSFREAEIVRAQQQYQQQQQQQPQQVKKSRRGPRSRSSQYRGVTFYRRTG
RWESHIWDCGKQVYLGGFDTAHAAARAYDRAAIKFRGVDADINFNLSDYEEDMKQMKSLSKEEFVHVLRRQSTGFSRGSSKYRGVTL
HKCGRWEARMGQFLGKKYIYLGLFDSEVEAARAYDKAAIKCNGREAVTNFEPSTYEGETISDPQSAGGQQDLDLNLGIATSSPKENER
MGGFQYHPYDMQDASKSQMDKSAPAIVGSSHFKAQPVTSEQAHLWNGVYSNFFPSYEERASGKRIDVGSSQGRPNWALQMHGQ
VGTTPMSMFTAAASSGFSTPATTASASPMSGPNPNTNTPNLPFASYSTPSTNTTQYYYQIRPPLPPP

>Tobacco_XP_016475254

MLDLNVSAVSVNSNSDETDPNNNTLFFKDEYSAGTSNTTASSSVVNAPVAGDEDSNSSSVHHGSKRAHHSLSTLNFSILKSDRAIEIED
DMSTDDVLTRQLFPVKNESQVQVQVQGTRIQGQSQCWLNLSVPESGGGAEMGINKPPTPAQQPVKKSRRGPRSRSSQYRGVTFYR
RTGRWESHIWDCGKQVYLGGFDTAHAAARAYDRAAIKFRGVDADINFNITDYEEDMEQMKNLSKEEFVQVLRRQSTGFSRGNSKYR
GVTLHKCGRWEARMGQFLGKKAYDKAAIKCNGREAVTNFEPSTYEREIKEDNKDGGSGQNLDLNLWISPPLAGPKRDEIGRNVPFNF
GAREMVIGRRLEIENSSTAPQGAPTASKLSLLTGVYPGFSPHNMEGAMAKVEATSSPGFPNWAWKIHSQGLVTPVPVFSSAASSGFS
TTTTLYPNTLLLPSNQLRLPTNSTFQHPPVTLNIS

Figure S1

D

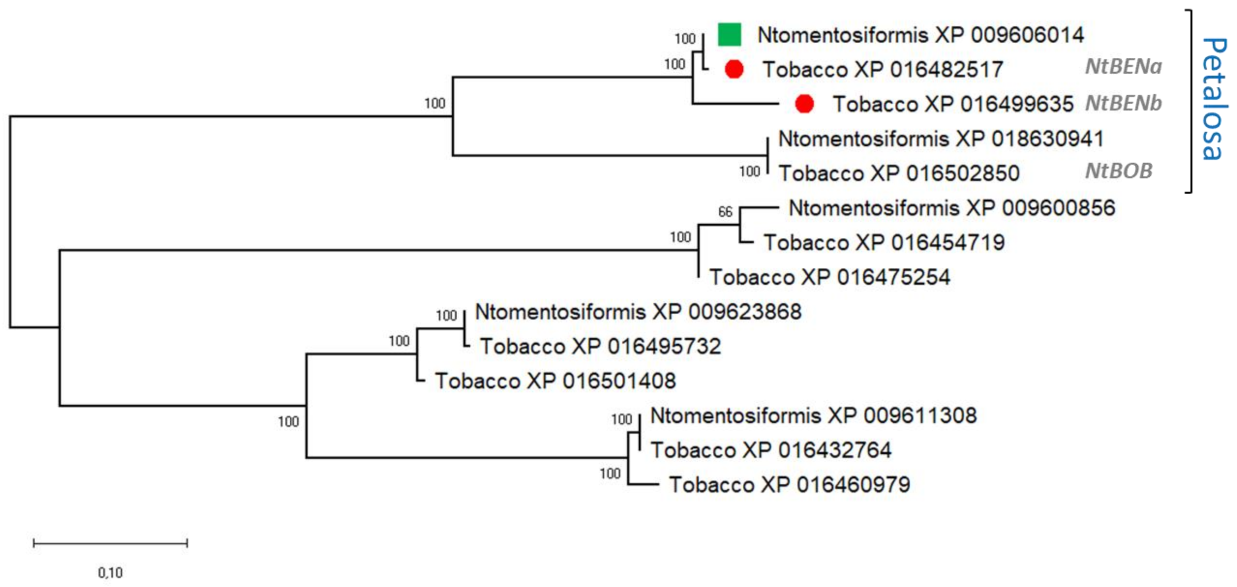

Figure S1. TOE-type peptides from different species. (A) Sequences used to generate the phylogenetic tree (Fig. 4). (B) *B. napus* TOE-type proteins. (C) *N. tabacum* TOE-type proteins. (D) Maximum likelihood tree illustrating the relationships between *N. tomentosiformis* and *N. tabacum* TOE-type proteins. A square indicates *N. tomentosiformis* BEN (compare to Fig. 4). Red dots designate sequences targeted for gene editing.

Figure S2

A

```
Prupe.6G242400     GAAAGAGCAACGGAAAAGAGAATGGATGTTAACTCCTTTCCAAATTGGGCATGGCAACTC
Dca21030_Gen       GAAAGGGCGGTAGAGAAGGTAGCAGAAAGTGATCCTTGGACGACCTGGGCACGGAAACTC
                   *****.**..  .**.***. *.  .**:.  *.*   * *   .*.*. ****** **.*****

Prupe.6G242400     CAAGGCCTTAATGGTGGAGCAACTCCAATGCCACTCTTCTCTGCTGCAGCATCATCAGGA
Dca21030_Gen       CAAG------------GAACTCAAACTGTACCCTTCTACGCTAATGCAGCATCATCAGGA
                   ****            **.*:..:.*:.*.**. ***:* **..****************

Prupe.6G242400     TT----------------------------------------------------------
Dca21030_Gen       TTAGACCTGGCAAAATGAAACCGAACCAGATAAACCGACTGTAATTGTTGGAATCGACAT
                   **

Prupe.6G242400     ------------------------------------------------------------
Dca21030_Gen       CCAACCGAACATCTGAAAAGTTTAACTTGAACTTAATCCGTAACCTGATATGACTTGCAC

Prupe.6G242400     ------------------------------------------------------------
Dca21030_Gen       TGAACTGAAACTGAACGAAATTCAACACATATCTGGAAAGACCCATAGTCGAATAAACTT

Prupe.6G242400     ------------------------------------------------------------
Dca21030_Gen       GAACTGTTTTAAATCCGAATTTATTTCACTCGAACCCGAAGACAACTGAACTGAAAAATA

Prupe.6G242400     ------------------------------------------------------------
Dca21030_Gen       ATCGATTCGACCCAAACCAAAAGAATATTTACCTCATATATAAATATATATATTAAAGGC

Prupe.6G242400     ------------------------------------------------------------
Dca21030_Gen       TACTTTATATGACAACTGTCATACAAGTTCAAAATCAACTTCTTAACTCTTTAACCAAGT

Prupe.6G242400     ------------------------------------------------------------
Dca21030_Gen       GTTTAAGAGTATTAATTGTTTTGATAATTGAAAATAATAAAAATGACTCCTATTTATAAC

Prupe.6G242400     ------------------------------------------------------------
Dca21030_Gen       ATAATATCACTTCATAACTAGGTAATCTTTCGATGTGGGATATTGTAACTATTTATACCA

Prupe.6G242400     ------------------------------------------------------------
Dca21030_Gen       AATTCATCACATTTACATTCTTATTTTTTCAATGTGGGACACAATAGAACAAGAGTATTC

Prupe.6G242400     ------------------------------------------------------------
Dca21030_Gen       CAAGTTAAAGTCGCATCTTAAAAACAATATTATAATTTAGTATGAGACGGATAAACTCAT

Prupe.6G242400     ------------------------------------------------------------
Dca21030_Gen       CGAAACACGAAGCTGTAAAATAATATAAAATCTAAGACCTTTAAACTTTTATTCCGTATT

Prupe.6G242400     ------------------------------------------------------------
Dca21030_Gen       TTGCAACATATTTATTTTTTCACATAACTCTTATATGCGAAAATTTAGAAGCACACAAGT

Prupe.6G242400     ------------------------------------------------------------
Dca21030_Gen       GGAAAATGAAATCAAAATTGAGGACGTTACAATTCTTTCATATATTATCCGAATGTGACC

Prupe.6G242400     ------------------------------------------------------------
Dca21030_Gen       CGATTCGAATAATATCTGACTTGATCATATCTCGACTCGATTATCAACTGATCTGACATT

Prupe.6G242400     ------------------------------------------------------------
Dca21030_Gen       AATCCGACTTGAACCCGAATTGAACCAAAATTATCTACAACCGATAGAGACTCGTAATCG

Prupe.6G242400     ------------------------------------------------------------
Dca21030_Gen       AAACAAAATCGAATTGAACTGAATTGAAATCGAAAATACAAATAAACCAAATGAAATCGA

Prupe.6G242400     ----------------------------------------------CCCTTCTTCAACAG
Dca21030_Gen       TCCGAAACAAACTGATCCGAAACCGATCGAAATGACCCGATTGCCACCTCTAATCAGGAT
                                                                 **  *.:***. *

Prupe.6G242400     CCACTTCACCACCAGCTGCTGTCACTCAACTTCATTTTCCTAACACAACTATTCTCCACC
Dca21030_Gen       TCCCTTCAATTGCAACTGCT-TCA-ACAGCTGCTTTTGGCCAACAACAATATTCTAACTC
                    *.*****. : **.***** *** :**.** *:***  * ****..*.******... *

Prupe.6G242400     ACCATTTTTCACCATCGACAGTCACCAACAACATACCCGGTTTCTATTGCAGGAGCTGA
Dca21030_Gen       AACATCGTTACATTACTCCTATCCTCTTTAAGACGCCATTTTTCTATCTAACAGCGTGA
                   *.***  **... ::* .*:.**. *:: ** * .**.  *******  .* ..  ***
```

Figure S2

B

Sequences	Scaffold	Score (Bits)	E Value	Max ident
DF342964.1	scaffold2131	1855	0.0	100%
DF340866.1	scaffold3	1832	0.0	100%
DF343639.1	scaffold2844	1827	0.0	100%
DF344517.1	scaffold3778	1827	0.0	100%
DF343163.1	scaffold2341	1825	0.0	100%
DF343106.1	scaffold2282	1816	0.0	99%
DF347580.1	scaffold7351	1786	0.0	99%
DF340959.1	scaffold96	1783	0.0	99%
DF341080.1	scaffold217	1779	0.0	99%
DF342876.1	scaffold2041	1777	0.0	99%
DF341545.1	scaffold684	1753	0.0	98%
DF344129.1	scaffold3363	1749	0.0	98%
DF341770.1	scaffold911	1742	0.0	98%
DF342221.1	scaffold1371	1720	0.0	98%
DF343341.1	scaffold2530	1716	0.0	98%
DF350304.1	scaffold11014	1716	0.0	98%
DF344846.1	scaffold4147	1712	0.0	97%
DF340878.1	scaffold15	1705	0.0	97%
DF341373.1	scaffold510	1698	0.0	97%
DF345773.1	scaffold5201	1687	0.0	97%
DF348201.1	scaffold8123	1677	0.0	97%
DF341602.1	scaffold742	1620	0.0	96%
DF341983.1	scaffold1130	1594	0.0	99%
DF341507.1	scaffold645	1544	0.0	95%
DF342706.1	scaffold1864	1487	0.0	97%
DF342208.1	scaffold1358	1450	0.0	99%
DF344202.1	scaffold3442	1424	0.0	97%
DF341118.1	scaffold255	1406	0.0	92%
DF342740.1	scaffold1900	1225	0.0	98%
DF346478.1	scaffold6000	1184	0.0	97%
DF342185.1	scaffold1334	1134	0.0	98%
DF352731.1	scaffold14784	1127	0.0	98%
DF346313.1	scaffold5810	1125	0.0	94%
DF345341.1	scaffold4708	1107	0.0	93%
DF341003.1	scaffold140	1099	0.0	94%
DF341058.1	scaffold195	1094	0.0	96%
DF340911.1	scaffold48	1092	0.0	93%
DF343116.1	scaffold2292	1085	0.0	97%
DF343852.1	scaffold3070	1079	0.0	94%
DF345148.1	scaffold4485	1062	0.0	94%
DF341345.1	scaffold482	1061	0.0	94%
DF341381.1	scaffold518	1051	0.0	93%
DF350569.1	scaffold11394	992	0.0	91%
DF349784.1	scaffold10260	893	0.0	96%
DF342835.1	scaffold1998	874	0.0	97%
DF350440.1	scaffold11207	863	0.0	96%
DF341207.1	scaffold344	846	0.0	98%
DF346531.1	scaffold6063	774	0.0	98%
DF341832.1	scaffold976	756	0.0	95%
DF347528.1	scaffold7288	712	0.0	97%
DF341351.1	scaffold488	710	0.0	98%
DF343980.1	scaffold3207	688	0.0	90%
DF342509.1	scaffold1664	660	0.0	91%

Figure S2

C

```
Caryophyllus_Pet      CGTGGAGCGACATCAATTCCATGTCCCTTCCCGTCTATCAGGTAATTACTGAGTATTCAA
Caryophyllus_WT       CGTGGAGCGACATCAATTCCATGTCCCTTCCCGTCTATCAGGTAATTACTGAGTATTCAA
Deltoides             CGTGGAGCGACATCAATTCCATGTCCCTTCCCGTCTATCAGGTAATTACCGAGTATTCAA
Superbus              CGTGGAGCGACATCAATTCCATGTCCCTTCCCGTCTATCAGGTAATTACTGAGTATTCAA
                      Dca21030 Exon 9

Caryophyllus_Pet      GCTTATAGACGGTTTTCTGTTGTAAGACATGTATCTTGTAATTTAAGACGAGAGAAATAT
Caryophyllus_WT       GCTTATAGACGGTTTTCTGTTGTAAGACATGTATCTTGTAATTTAAGACGAGAGAAATAT
Deltoides             GCTTATAAACAGTTTTCTGTTGTAAGACATGTATCTG-TAATTTAAGACGAGAG-AATGT
Superbus              GCTTATAGACGGTTTTCTGTTGTAAGACATGTATCTG-TAATTTAAGACGAGAG-AATAT

Caryophyllus_Pet      CCTCGGAAAAATGACAGGAAAGGGCGGTAGAGAAGGTAGCAGAAAGTGATCCTTGGACGA
Caryophyllus_WT       CCTCGGAAAAATGACAGGAAAGGGCGGTAGAGAAGGTAGCAGAAAGTGATCCTTGGACGA
Deltoides             CCTCGGAAAAATGACAGGAAAGGGCGGTAGAGAAGGTAGCAGAAAGTGATCCTTGGACGA
Superbus              CCTGGGAAAAATGACAGGAAAGGGCGGTAGAGAAGGTAGCAGAAAGTGATCCTTGGACGA

Caryophyllus_Pet      CCTGGGCACGGAAACTCCAAGGAACTCAAACTGTACCCTTCTACGCTAATGCAGCATCAT
Caryophyllus_WT       CCTGGGCACGGAAACTCCAAGGAACTCAAACTGTACCCTTCTACGCTAATGCAGCATCAT
Deltoides             CCTGGGCACGGAAACTCCAAGGAACTCAAACTGTACCCTTCTACGCTAATGCAGCATCAT
Superbus              CCTGGGCACGGAAACACCAAGGAACTCAAACTGTACCCTTCTACGCTAATGCAGCATCAT

Caryophyllus_Pet      CAGGATTAGACCTGGCAAAATGAAACCGAACCAGATAAACCGACTGTAATTGTTGGAATC
Caryophyllus_WT       CAGGATTCCCTTCAATTGCAACTGCTTCAACAGCTGCTTTTGGCCAACAACAATATTCTA
Deltoides             CAGGATTCCCTTCAATTGCAACTGCTTCAACAGCTGCTTTTGGCCAACAACAATATTCTA
Superbus              CAGGATTCCCTTCAATTGCAACTGCTTCAACAGCTGCTTTTGGCCAACTACAATATTCTA

Caryophyllus_Pet      GACATCCAACCGAACATCTGAAAAGTTTAACTTGAACTTAATCCGTAACCTGATA
Caryophyllus_WT       ACTCAACATCGTTACATTACTCCTATCCTCTTTAAGACGCCATTT-TTCTATCTA
Deltoides             ACTCAACATCGTTACATTACTCCTATCCTCTTTAAGACGCCATTTATTCCATCTA
Superbus              ACTCAACATCGTTACATTACTCCTATCCTCTTTAAGACGCCATTT-TTCTATCTA
                      Dca21030 Exon 10
```

D

```
                                         miR172 binding site
Peach(Prupe.6G242400)      CCACUCUUCUCUGCUGCAGCAUCAUCAGGAUUCCCUUCUUCAACAGC
Rose (XM_024326925)        CCACUCUUCUCUGCGGCAGCAUCAUCAGGAUUCCCUUCUUCAACAAC
Petunia (KU096996)         CCAUUCUUCUCUACUGCAGCAUCAUCAGGAUUCGUAAAUUCAGCAGU
Dianthus WT(Dca21030.2)    CCCUUCUACGCUAAUGCAGCAUCAUCAGGAUUCCCUUCAAUUGCAAC
Dianthus Pet(Dca21030.1)   CCCUUCUACGCUAAUGCAGCAUCAUCAGGAUUAGACCUGGCAAAAUG
```

Figure S2. **Analysis of the *Dianthus pet* allele.** (A) Sequence alignment between the 10th exon of the Prupe.6G242400 CDS and 1257bp of *Dianthus* genomic DNA, starting from the putative 10th exon of Dca21030.1. Prupe.6G242400 miR172 binding site is highlighted in grey, while arrowheads indicate the boundaries of a putative 1004 bp insertion in the *Dianthus* sequence. (B) BLAST hits of the insertion sequence against the *D. caryophyllus* reference genome database DCA_r1.0 GenBank assembly [GCA_000512335.1] using as a query the 1004 bp insertion from Fig. 1A (C) Sequence alignment of 3' portion of *Dca21030* alleles amplified from DF *D. caryophyllus* variety 'Widecombe fair' and single flower accessions *D. superbus* 'Primadonna' and *D. deltoides* 'Flashing lights'. CDS exons sequences are highlighted in gray. 'Widecombe fair' harbours both wild-type and mutated (*pet,* bold and italics) allele. miR172 binding sites are underlined and STOP codons are boxed. (D) Comparison of putative miR172 binding sites in *PET* transcripts from different species, for *Dianthus* both wild-type and *pet* alleles are indicated.

Figure S3

A

```
Dca21030.2        ATGGTGGATCTTAATCTCACCTTACACCCACCCGATTCCACTTCTGGTTACGACACCGTC
Dca21030.1_pet    ATGGTGGATCTTAATCTCACCTTACACCCACCCGATTCCACTTCTGGTTACGACACCGTC
                  ************************************************************

Dca21030.2        TCAAACGCGCCCACTTTTAACTTTGAAGGTATTCTTGGGACTTCCAACTGTGGTTACTTG
Dca21030.1_pet    TCAAACGCGCCCACTTTTAACTTTGAAGGTATTCTTGGGACTTCCAACTGTGGTTACTTG
                  ************************************************************

Dca21030.2        GGCAATGATCGCGGGGTTTACGGGCCGAGTCGTGTCGGGTATGTTGTTATGGGTCAGGGT
Dca21030.1_pet    GGCAATGATCGCGGGGTTTACGGGCCGAGTCGTGTCGGGTATGTTGTTATGGGTCAGGGT
                  ************************************************************

Dca21030.2        TTGAGTCAGGTGAAGAAGAGTAGGAGGGGCCCACGGTCTAGGAGTTCACAGTATCGTGGA
Dca21030.1_pet    TTGAGTCAGGTGAAGAAGAGTAGGAGGGGCCCACGGTCTAGGAGTTCACAGTATCGTGGA
                  ************************************************************

Dca21030.2        GTTACTTTCTACCGTAGGACTGGCAGATGGGAGTCTCATATCTGGGACAATGGAAAACAG
Dca21030.1_pet    GTTACTTTCTACCGTAGGACTGGCAGATGGGAGTCTCATATCTGGGACAATGGAAAACAG
                  ************************************************************

Dca21030.2        GTGTACCTAGGAGGATTTGATACTGCTCATGCTGCTGCAAGGGCATATGATCGCGCTGCC
Dca21030.1_pet    GTGTACCTAGGAGGATTTGATACTGCTCATGCTGCTGCAAGGGCATATGATCGCGCTGCC
                  ************************************************************

Dca21030.2        ATCAAGTTTCGAGGAGTTGATGCAGATATCAATTTTGATGTCAGTGATTATGAAGACGAT
Dca21030.1_pet    ATCAAGTTTCGAGGAGTTGATGCAGATATCAATTTTGATGTCAGTGATTATGAAGACGAT
                  ************************************************************

Dca21030.2        CTTAAACAAATGAGTAACTTGACAAAGGAAGAATTTGTGCATCTTCTTCGTCGTCAAAGC
Dca21030.1_pet    CTTAAACAAATGAGTAACTTGACAAAGGAAGAATTTGTGCATCTTCTTCGTCGTCAAAGC
                  ************************************************************

Dca21030.2        ACTGGCTTCTCCAGAGGAAGCTCCAAGTACAGGGGCGTGACGCTGCACAAATGTGGTCGA
Dca21030.1_pet    ACTGGCTTCTCCAGAGGAAGCTCCAAGTACAGGGGCGTGACGCTGCACAAATGTGGTCGA
                  ************************************************************

Dca21030.2        TGGGAGGCTCGTATGGGACAACTCTTAGGAAAAAAGTACATTTATCTCGGCTTGTTTGAC
Dca21030.1_pet    TGGGAGGCTCGTATGGGACAACTCTTAGGAAAAAAGTACATTTATCTCGGCTTGTTTGAC
                  ************************************************************

Dca21030.2        AGTGAACTCGAAGCTGCAAGAGCCTACGACAAGGCAGCAATTCAGTGCAATGGAAAGGAA
Dca21030.1_pet    AGTGAACTCGAAGCTGCAAGAGCCTACGACAAGGCAGCAATTCAGTGCAATGGAAAGGAA
                  ************************************************************

Dca21030.2        GCCGTCACAAACTTTGATCCAACCACATATGGAGACGAGCTAATGGACGAAACGCCTTTT
Dca21030.1_pet    GCCGTCACAAACTTTGATCCAACCACATATGGAGACGAGCTAATGGACGAAACGCCTTTT
                  ************************************************************

Dca21030.2        GGAGGTGGACCGAATTCTCTTGATTTGAACTTGGGAATCGGCAATGCCTCCTCCGCTGAT
Dca21030.1_pet    GGAGGTGGACCGAATTCTCTTGATTTGAACTTGGGAATCGGCAATGCCTCCTCCGCTGAT
                  ************************************************************

Dca21030.2        GGGCACGTGAAAACCCACAATTTAGACGGTATGCTAAACATGAGAGCAAAGAGTCAAAAT
Dca21030.1_pet    GGGCACGTGAAAACCCACAATTTAGACGGTATGCTAAACATGAGAGCAAAGAGTCAAAAT
                  ************************************************************

Dca21030.2        CGGATTTCAGCAGTGGAAATGCCTCCGATTTGTCACGGTCACAGATACCTTTCTGAGCTC
Dca21030.1_pet    CGGATTTCAGCAGTGGAAATGCCTCCGATTTGTCACGGTCACAGATACCTTTCTGAGCTC
                  ************************************************************

Dca21030.2        CCTACTACGTGGAGCGACATCAATTCCATGTCCCTTCCCGTCTATCAGGAAAGGGCGGTA
Dca21030.1_pet    CCTACTACGTGGAGCGACATCAATTCCATGTCCCTTCCCGTCTATCAGGAAAGGGCGGTA
                  ************************************************************

Dca21030.2        GAGAAGGTAGCAGAAAGTGATCCTTGGACGACCTGGGCACGGAAACTCCAAGGAACTCAA
Dca21030.1_pet    GAGAAGGTAGCAGAAAGTGATCCTTGGACGACCTGGGCACGGAAACTCCAAGGAACTCAA
                  ************************************************************

                                     miR172 binding
Dca21030.2        ACTGTACCCTTCTACGCTAATGCAGCATCATCAGGATTCCCTTCAATTGCAACTGCTTCA
Dca21030.1_pet    ACTGTACCCTTCTACGCTAATGCAGCATCATCAGGATTAGACCTGGCAAAATGA------
                  **************************************. .   .. :..*: :

Dca21030.2        ACAGCTGCTTTTGGCCAACAACAATATTCTAACTCAACATCGTTACATTACTCCTATCCT
Dca21030.1_pet    ------------------------------------------------------------

Dca21030.2        CTTTAA
Dca21030.1_pet    ------
```

Figure S3

B

```
                            NLS                                       AP2-R1
Dca21030.2        MVDLNLTLHPPDSTSGYDTVSNAPTFNFEGILGTSNCGYLGNDRGVYGPSRVGYVVMGQG
Dca21030.1_pet    MVDLNLTLHPPDSTSGYDTVSNAPTFNFEGILGTSNCGYLGNDRGVYGPSRVGYVVMGQG

                       NLS                                       AP2-R1
Dca21030.2        LSQVKKSRRGPRSRSSQYRGVTFYRRTGRWESHIWDNGKQVYLGGFDTAHAAARAYDRAA
Dca21030.1_pet    LSQVKKSRRGPRSRSSQYRGVTFYRRTGRWESHIWDNGKQVYLGGFDTAHAAARAYDRAA

                                                          Linker
Dca21030.2        IKFRGVDADINFDVSDYEDDLKQMSNLTKEEFVHLLRRQSTGFSRGSSKYRGVTLHKCGR
Dca21030.1_pet    IKFRGVDADINFDVSDYEDDLKQMSNLTKEEFVHLLRRQSTGFSRGSSKYRGVTLHKCGR

                        AP2-R2
Dca21030.2        WEARMGQLLGKKYIYLGLFDSELEAARAYDKAAIQCNGKEAVTNFDPTTYGDELMDETPF
Dca21030.1_pet    WEARMGQLLGKKYIYLGLFDSELEAARAYDKAAIQCNGKEAVTNFDPTTYGDELMDETPF

                         EAR
Dca21030.2        GGGPNSLDLNLGIGNASSADGHVKTHNLDGMLNMRAKSQNRISAVEMPPICHGHRYLSEL
Dca21030.1_pet    GGGPNSLDLNLGIGNASSADGHVKTHNLDGMLNMRAKSQNRISAVEMPPICHGHRYLSEL

                                                                 (miR172)
Dca21030.2        PTTWSDINSMSLPVYQERAVEKVAESDPWTTWARKLQGTQTVPFYANAASSGFPSIATAS
Dca21030.1_pet    PTTWSDINSMSLPVYQERAVEKVAESDPWTTWARKLQGTQTVPFYANAASSGLDLAK

Dca21030.2        TAAFGQQQYSNSTSLHYSYPL
Dca21030.1_pet
```

Figure S3

C

```
Ph_PETALOSA         ATGTTTGATCTTAATCTTAGTTGTGATGATGTTCCAGAAGAATCTGATCAGCTCAATGAA
Ph_PETALOSA_pet     ATGTTTGATCTTAATCTTAGTTGTGATGATGTTCCAGAAGAATCTGATCAGCTCAATGAA
                    ************************************************************

Ph_PETALOSA         CTTTCAGTTACTCAAATGGAGAATTCAAGAACTTCCAGTACTATTGGTGATGATGATAAT
Ph_PETALOSA_pet     CTTTCAGTTACTCAAATGGAGAATTCAAGAACTTCCAGTACTATTGGTGATGATGATAAT
                    ************************************************************

Ph_PETALOSA         TCATGTTCAGATCATATGTCAAATTGTTATACATTTGATATACTGAAAGCCAATAGGAAA
Ph_PETALOSA_pet     TCATGTTCAGATCATATGTCAAATTGTTATACATTTGATATACTGAAAGCCAATAGGAAA
                    ************************************************************

Ph_PETALOSA         GAAAGTGAAAATCTTGGTAGTTTTGTGACAAAAGAGTTGTTACCATTGAGTTTGAATGAT
Ph_PETALOSA_pet     GAAAGTGAAAATCTTGGTAGTTTTGTGACAAAAGAGTTGTTACCATTGAGTTTGAATGAT
                    ************************************************************

Ph_PETALOSA         CAGAGCATGGTTTTAGCTCAACAGCAGAGGTTACAGGTGATCAAGAAGAGTAGAAGAGGA
Ph_PETALOSA_pet     CAGAGCATGGTTTTAGCTCAACAGCAGAGGTTACAGGTGATCAAGAAGAGTAGAAGAGGA
                    ************************************************************

Ph_PETALOSA         CCAAGGTCTCGAAGTTCACAATATCGTGGAGTTACATTCTATCGAAGAACTGGAAGATGG
Ph_PETALOSA_pet     CCAAGGTCTCGAAGTTCACAATATCGTGGAGTTACATTCTATCGAAGAACTGGAAGATGG
                    ************************************************************

Ph_PETALOSA         GAATCTCATATATGGGATGGTGGGAAACAAGTCTACTTGGGGGGTTTTGACACTGCACAT
Ph_PETALOSA_pet     GAATCTCATATATGGGATGGTGGGAAACAAGTCTACTTGGGGGGTTTTGACACTGCACAT
                    ************************************************************

Ph_PETALOSA         GCTGCTGCTAGGGCATATGACCGTGCTGCGATTAAGTTCCGAGGATTTGATGCAGATATC
Ph_PETALOSA_pet     GCTGCTGCTAGGGCATATGACCGTGCTGCGATTAAGTTCCGAGGACTTGATGCAGATATC
                    ********************************************* **************

Ph_PETALOSA         AATTTTAACGTTAGTGATTATGAAGAAGATCTGAAGCAGATGAAGAACTTTTCCAAAGAA
Ph_PETALOSA_pet     AATTTTAACGTTAGTGATTATGAAGAAGATCTGAAGCAGATGAAGAACTTTTCCAAAGAA
                    ************************************************************

Ph_PETALOSA         GAGTTTCTGCACATACTTCGTCGTCAGAGCACTGGTTTCTCTAGAGGAAGTTCGAAGTTC
Ph_PETALOSA_pet     GAGTTTCTGCACATACTTCGTCGTCAGAGCACTGGTTTCTCTAGAGGAAGTTCGAAGTTC
                    ************************************************************

Ph_PETALOSA         AGGGGAGTCACTCTGCATAAATGTGGACGATGGGAAGCGCGGATGGGCCAGCTTCTTGGA
Ph_PETALOSA_pet     AGGGGAGTCACTCTGCATAAATGTGGACGATGGGAAGCGCGGATGGGCCAGCTTCTTGGA
                    ************************************************************

Ph_PETALOSA         AAGAAGTATATCTATCTTGGACTATTTGACAGTGAGATAGAAGCTGCAAGGGCATATGAT
Ph_PETALOSA_pet     AAGAAGTATATCTATCTTGGACTATTTGACAGTGAGATAGAAGCTGCAAGGGCATATGAT
                    ************************************************************

Ph_PETALOSA         AAGGCTGCTATAAAAAGCAATGGGAGAGAAGCAGTCACCAATTTTGAGCTGAGCACATAT
Ph_PETALOSA_pet     AAGGCTGCTATAAAAAGCAATGGGAGAGAAGCAGTCACCAATTTTGAGCTGAGCACATAT
                    ************************************************************

Ph_PETALOSA         GAAGGGGTATTAAGTTCTGAGACTGCTGATACTGGAGGCACAAGTCATAATCTTGACCTG
Ph_PETALOSA_pet     GAAGGGGTATTAAGTTCTGAGACTGCTGATACTGGAGGCACAAGTCATAATCTTGATCTG
                    ******************************************************** ***

Ph_PETALOSA         AGATTGGGCATATCTCCCTCTTCCTGTGCTGACAATCAACATGGAAATACCAGCCAAATG
Ph_PETALOSA_pet     AGATTGGGCATATCTCCCTCTTCCTGTGCTGACAATCAACATGGAAATACCAGCCAAATG
                    ************************************************************

Ph_PETALOSA         GGAATCTCTCAGTGCCGGCCTGGCTCAAATGGTTTACCTGAACATAGAGAAGTCTTGAGC
Ph_PETALOSA_pet     GGAATCTCTCAGTGCCGGCCTGGCTCAAATGGTTTACCTGAACATAGAGAAGTCTTGAGC
                    ************************************************************

Ph_PETALOSA         TCTGCTTCTACTACACCAAGAAGTATGCTGCTTCATGGTCAGCATATGCTAGATCAGCAC
Ph_PETALOSA_pet     TCTGCTTCTACTACACCAAGAAGTATGCTGCTTCATGGTCAGCATATGCTAGATCAGCAC
                    ************************************************************

Ph_PETALOSA         CCCCTCCATTGGAATGGACCGAATGACAATCTCTTTCCCACATTTAAGGGAACAGCAATA
Ph_PETALOSA_pet     CCCCTCCATTGGAATGGACCGAATGACAATCTCTTTCCCACATTTAAGGGAACAGCAATA
                    ************************************************************

Ph_PETALOSA         GAGAAGGGCTTGGAAGTTGACACCTTATCCAAATGGATG-CGGCAAGACCAGAATTCTTA
Ph_PETALOSA_pet     GAGAAGGGCTTGGAAGTTGACACTGTAACAACTAAATTCGCGTCAAGAAAATAA------
                    ***********************  **:*.*.::..:*  ** *****..* **

                                                           miR172 binding
Ph_PETALOSA         TGGTGGAAGTCCTACAGCTCCATTCTTCTCTACTGCAGCATCATCAGGATTCGTAAATTC
Ph_PETALOSA_pet     ------------------------------------------------------------


Ph_PETALOSA         AGCAATCAGTGGACCTTCAGCTGTTGTTCATCAACTGCACTTTCCTAGTAGAGCACTACC
Ph_PETALOSA_pet     ------------------------------------------------------------


Ph_PETALOSA         TTATCACTATTCACCATCCCTCACAAATGAAACCTTCACATTATTATTGCAGGAGCTGAA
Ph_PETALOSA_pet     ------------------------------------------------------------


Ph_PETALOSA         ATAG
Ph_PETALOSA_pet     ----
```

Figure S3

D

```
                                                                                  NLS
Ph_PETALOSA          MFDLNLSCDDVPEESDQLNELSVTQMENSRTSSTIGDDDNSCSDHMSNCYTFDILKANRK
Ph_PETALOSA_pet      MFDLNLSCDDVPEESDQLNELSVTQMENSRTSSTIGDDDNSCSDHMSNCYTFDILKANRK

                                                                   NLS
Ph_PETALOSA          ESENLGSFVTKELLPLSLNDQSMVLAQQQRLQVIKKSRRGPRSRSSQYRGVTFYRRTGRW
Ph_PETALOSA_pet      ESENLGSFVTKELLPLSLNDQSMVLAQQQRLQVIKKSRRGPRSRSSQYRGVTFYRRTGRW

                      AP2-R1
Ph_PETALOSA          ESHIWDGGKQVYLGGFDTAHAAARAYDRAAIKFRGFDADINFNVSDYEEDLKQMKNFSKE
Ph_PETALOSA_pet      ESHIWDGGKQVYLGGFDTAHAAARAYDRAAIKFRGLDADINFNVSDYEEDLKQMKNFSKE

                      Linker                                  AP2-R2
Ph_PETALOSA          EFLHILRRQSTGFSRGSSKFRGVTLHKCGRWEARMGQLLGKKYIYLGLFDSEIEAARAYD
Ph_PETALOSA_pet      EFLHILRRQSTGFSRGSSKFRGVTLHKCGRWEARMGQLLGKKYIYLGLFDSEIEAARAYD

                                                          EAR
Ph_PETALOSA          KAAIKSNGREAVTNFELSTYEGVLSSETADTGGTSHNLDLRLGISPSSCADNQHGNTSQM
Ph_PETALOSA_pet      KAAIKSNGREAVTNFELSTYEGVLSSETADTGGTSHNLDLRLGISPSSCADNQHGNTSQM

Ph_PETALOSA          GISQCRPGSNGLPEHREVLSSASTTPRSMLLHGQHMLDQHPLHWNGPNDNLFPTFKGTAI
Ph_PETALOSA_pet      GISQCRPGSNGLPEHREVLSSASTTPRSMLLHGQHMLDQHPLHWNGPNDNLFPTFKGTAI

                                                        (miR172)
Ph_PETALOSA          EKGLEVDTLSKWMRQDQNSYGGSPTAPFFSTAASSGFVNSAISGPSAVVHQLHFPSRALP
Ph_PETALOSA_pet      EKGLEVDTVTTKFASRK

Ph_PETALOSA          YHYSPSLTNETFTLLLQELK
Ph_PETALOSA_pet
```

Figure S3

E

```
Rr_PETALOSA_wt       ATGCTAGATCTCAACCTCAACTTCTACACTCCCGAAATGGAGACCTCCGCCGCGAGCAGC
Rr_PETALOSA_pet      ATGCTAGATCTCAACCTCAACTTCTACACTCCCGAAATGGAGACCTCCGCCGCGAGCAGC
                     ************************************************************

Rr_PETALOSA_wt       TTCAACTCCTCCTCCAACGACTCCTCCTCCTCCGCCACCCTCAACTTCCTCGACCTCCAA
Rr_PETALOSA_pet      TTCAACTCCTCCTCCAACGACTCCTCCTCCTCCGCCACCCTCAACTTCCTCGACCTCCAA
                     ************************************************************

Rr_PETALOSA_wt       AACGACGTCGCCGCCCCCCCTTACGACGACGACGACGACGCCCATCATCATAACTACTTC
Rr_PETALOSA_pet      AACGACGTCGCCGCCCCCCCTTACGACGACGACGACGACGCCCATCATCATAACTACTTC
                     ************************************************************

Rr_PETALOSA_wt       GACGGCCGCACCATCCAGCTCTTCCCTCCTCAACCCCAGCCCCCACGGTCTTCGACGACC
Rr_PETALOSA_pet      GACGGCCGCACCATCCAGCTCTTCCCTCCTCAACCCCAGCCCCCACGGTCTTCGACGACC
                     ************************************************************

Rr_PETALOSA_wt       AAGCAGTGGCTGGGGCTCTCCTCCGGCGGAGCACCGCAGCGTTACTGTGCTCCTCCTCCT
Rr_PETALOSA_pet      AAGCAGTGGCTGGGGCTCTCCTCCGGCGGAGCACCGCAGCGTTACTGTGCTCCTCCTCCT
                     ************************************************************

Rr_PETALOSA_wt       CCGCCGCTCGTGGAGCAGAGGATCGTGCCTCTCCCGGAGAAGGTCAAGAAGAGCCGCCGG
Rr_PETALOSA_pet      CCGCCGCTCGTGGAGCAGAGGATCGTGCCTCTCCCGGAGAAGGTCAAGAAGAGCCGCCGG
                     ************************************************************

Rr_PETALOSA_wt       GGACCGAGGTCCCGGAGCTCGCAGTATCGCGGCGTCACGTTTTATCGGAGGACCGGGAGA
Rr_PETALOSA_pet      GGACCGAGGTCCCGGAGCTCGCAGTATCGCGGCGTCACGTTTTATCGGAGGACCGGGAGA
                     ************************************************************

Rr_PETALOSA_wt       TGGGAATCGCATATTTGGGATAAGGGGAAGCAGGTGTACTTGGGTGGATTTGACACTGCG
Rr_PETALOSA_pet      TGGGAATCGCATATTTGGGATAAGGGGAAGCAGGTGTACTTGGGTGGATTTGACACTGCG
                     ************************************************************

Rr_PETALOSA_wt       CATTCTGCAGCTAGAGCTTATGATCGAGCTGCGATAAAGTTCCGTGGAATTGAGGCTGAT
Rr_PETALOSA_pet      CATTCTGCAGCTAGAGCTTATGATCGAGCTGCGATAAAGTTCCGTGGAATTGAGGCTGAT
                     ************************************************************

Rr_PETALOSA_wt       ATTAATTTCAATGTTAGTGATTATGATGAGGATATTAAGCAGATGAGTAAATTTACAAAG
Rr_PETALOSA_pet      ATTAATTTCAATGTTAGTGATTATGATGAGGATATTAAGCAGATGAGTAAATTTACAAAG
                     ************************************************************

Rr_PETALOSA_wt       GAAGAGTTTGTGCATATCCTGCGTCGCCAGAGCACCGGATTCGCCAGAGGAAGCTCGAAA
Rr_PETALOSA_pet      GAAGAGTTTGTGCATATCCTGCGTCGCCAGAGCACCGGATTCGCCAGAGGAAGCTCGAAA
                     ************************************************************

Rr_PETALOSA_wt       TACAGGGGAGTCACGTTGCACAAATGTGGCCGCTGGGAAGCTCGTATGGGCCAGTTGCTG
Rr_PETALOSA_pet      TACAGGGGAGTCACGTTGCACAAATGTGGCCGCTGGGAAGCTCGTATGGGCCAGTTGCTG
                     ************************************************************

Rr_PETALOSA_wt       GGCAAGAAGTATATATACCTTGGCCTATTTGATAGCGAGATTGAAGCTGCAAGAGCATAT
Rr_PETALOSA_pet      GGCAAGAAGTATATATACCTTGGCCTATTTGATAGCGAGATTGAAGCTGCAAGAGCATAT
                     ************************************************************

Rr_PETALOSA_wt       GACCAGGCTGCCATCAAATGCCATGGAAGGGAAGCGGTCACCAACTTTAATCCAAGCTTA
Rr_PETALOSA_pet      GACCAGGCTGCCATCAAATGCCATGGAAGGGAAGCGGTCACCAACTTTAATCCAAGCTTA
                     ************************************************************

Rr_PETALOSA_wt       TATGATGGGGAGATAATAATGGCTGAGGCCAACAATGCAGGCATCGATGATAGTCTTGAT
Rr_PETALOSA_pet      TATGATGGGGAGATAATAATGGCTGAGGCCAACAATGCAGGCATCGATGATAGTCTTGAT
                     ************************************************************

Rr_PETALOSA_wt       CTAAACTTGGGAATTGCTCCCCCCTTGGCTTCTGACTGTCCAAATGAGAACATCAACTTG
Rr_PETALOSA_pet      CTAAACTTGGGAATTGCTCCCCCCTTGGCTTCTGACTGTCCAAATGAGAACATCAACTTG
                     ************************************************************

Rr_PETALOSA_wt       AGCAGCTTTCCTTTTCAGCTAGGCTGCAATGACATTCCTGTTCACATGACAGCAAGGAAT
Rr_PETALOSA_pet      AGCAGCTTTCCTTTTCAGCTAGGCTGCAATGACATTCCTGTTCACATGACAGCAAGGAAT
                     ************************************************************

Rr_PETALOSA_wt       GAGAATTCTGTTCCAGGACTTCCAGCAACTATGAGGACTCAACTGCCTCATGGCTCAATG
Rr_PETALOSA_pet      GAGAATTCTGTTCCAGGACTTCCAGCAACTATGAGGACTCAACTGCCTCATGGCTCAATG
                     ************************************************************

Rr_PETALOSA_wt       GTGGCAACTACAGAGCCTCCTCTAATGAGCAGCATGACCTCCAACTTCTTTCCCATCCAT
Rr_PETALOSA_pet      GTGGCAACTACAGAGCCTCCTCTAATGAGCAGCATGACCTCCAACTTCTTTCCCATCCAT
                     ************************************************************

Rr_PETALOSA_wt       ATGGAAAGAGCAATGGAGAAGAGTATGGATGCTAACTCCTTCCCTAACTGGGCATGGCAA
Rr_PETALOSA_pet      ATGGTAATTTATTTTCAGTTCTAA------------------------------------
                     ****:** : .::*  **:: :.:

                                                                   miR172 binding
Rr_PETALOSA_wt       ATGCAAGGCCTTAATGATGGAGCAACTCCAGTGCCACTCTTCTCTGCGGCAGCATCATCA
Rr_PETALOSA_pet      ------------------------------------------------------------

Rr_PETALOSA_wt       GGATTCCCTTCTTCAACAACAGAATCATCAGCTCCGATCCCTCCACTTCATTTTCCCAAC
Rr_PETALOSA_pet      ------------------------------------------------------------

Rr_PETALOSA_wt       ACATCATTTCTCCAGCGCCACTTTTCACCATCGTTCACCAACAAAACACCCCATTTCTAT
Rr_PETALOSA_pet      ------------------------------------------------------------

Rr_PETALOSA_wt       TGCAGGAGCTGA
Rr_PETALOSA_pet      ------------
```

Figure S3

F

```
                        EAR
Rr_PETALOSA_wt        MLDLNLNFYTPEMETSAASSFNSSSNDSSSSATLNFLDLQNDVAAPPYDDDDDAHHHNYF
Rr_PETALOSA_pet       MLDLNLNFYTPEMETSAASSFNSSSNDSSSSATLNFLDLQNDVAAPPYDDDDDAHHHNYF

                                                                           NLS
Rr_PETALOSA_wt        DGRTIQLFPPQPQPPRSSTTKQWLGLSSGGAPQRYCAPPPPPLVEQRIVPLPEKVKKSRR
Rr_PETALOSA_pet       DGRTIQLFPPQPQPPRSSTTKQWLGLSSGGAPQRYCAPPPPPLVEQRIVPLPEKVKKSRR

                                                AP2-R1
Rr_PETALOSA_wt        GPRSRSSQYRGVTFYRRTGRWESHIWDKGKQVYLGGFDTAHSAARAYDRAAIKFRGIEAD
Rr_PETALOSA_pet       GPRSRSSQYRGVTFYRRTGRWESHIWDKGKQVYLGGFDTAHSAARAYDRAAIKFRGIEAD

                                          Linker
Rr_PETALOSA_wt        INFNVSDYDEDIKQMSKFTKEEFVHILRRQSTGFARGSSKYRGVTLHKCGRWEARMGQLL
Rr_PETALOSA_pet       INFNVSDYDEDIKQMSKFTKEEFVHILRRQSTGFARGSSKYRGVTLHKCGRWEARMGQLL

                          AP2-R2                                              EAR
Rr_PETALOSA_wt        GKKYIYLGLFDSEIEAARAYDQAAIKCHGREAVTNFNPSLYDGEIIMAEANNAGIDDSLD
Rr_PETALOSA_pet       GKKYIYLGLFDSEIEAARAYDQAAIKCHGREAVTNFNPSLYDGEIIMAEANNAGIDDSLD

Rr_PETALOSA_wt        LNLGIAPPLASDCPNENINLSSFPFQLGCNDIPVHMTARNENSVPGLPATMRTQLPHGSM
Rr_PETALOSA_pet       LNLGIAPPLASDCPNENINLSSFPFQLGCNDIPVHMTARNENSVPGLPATMRTQLPHGSM

                                                                          (miR172)
Rr_PETALOSA_wt        VATTEPPLMSSMTSNFFPIHMERAMEKSMDANSFPNWAWQMQGLNDGATPVPLFSAAASS
Rr_PETALOSA_pet       VATTEPPLMSSMTSNFFPIHMVIYFQF

Rr_PETALOSA_wt        GFPSSTTESSAPIPPLHFPNTSFLQRHFSPSFTNKTPHFYCRS
Rr_PETALOSA_pet
```

G

```
Rr_PETALOSA_wt          MLDLNLNFYTPEMETSAASSFNSSSNDSSSSATLNFLDLQNDVAAPPYDDDDDAHHHNYF
Rosa_XP_024186592       MLDLNLNFYTPEMETSAASSFNDSSNDSSSSATLNFLDLQNDVAAPPYDDAD---HHDYF
                        **********************.*************************** *   **:**

Rr_PETALOSA_wt          DGRTIQLFPPQPQPP-RSSTTKQWLGLSSGGAPQRYCAPPPPP-LVEQRIVPLPEKVKKS
Rc_XP_024186592         DGRTIQLFPPQPQPPLRSSTTKQWLGLSSGGAPQRYSAPPPPPPLAEQRIVPLPEKVKKS
                        *************** ********************.****** *.**************

Rr_PETALOSA_wt          RRGPRSRSSQYRGVTFYRRTGRWESHIWDKGKQVYLGGFDTAHSAARAYDRAAIKFRGIE
Rc_XP_024186592         RRGPRSRSSQYRGVTFYRRTGRWESHIWDNGKQVYLGGFDTAHSAARAYDRAAIKFRGTE
                        *****************************:**************************** *

Rr_PETALOSA_wt          ADINFNVSDYDEDIKQMSKFTKEEFVHILRRQSTGFARGSSKYRGVTLHKCGRWEARMGQ
Rc_XP_024186592         ADINFNVSDYDEDIKQMSKFTKEEFVHILRRQSTGFARGSSKYRGVTLHKCGRWEARMGQ
                        ************************************************************

Rr_PETALOSA_wt          LLGKKYIYLGLFDSEIEAARAYDQAAIKCHGREAVTNFNPSLYDGEIIMAEANNAGIDDS
Rc_XP_024186592         LLGKKYIYLGLFDSEIEAARAYDQAAIKCHGREAVTNFNPSLYDGEI-MAEANNAGIDDS
                        *********************************************** ************

Rr_PETALOSA_wt          LDLNLGIAPPLASDCPNENINLSSFPFQLGCNDIPVHMTARNENSVPGLPATMRTQLPHG
Rc_XP_024186592         LDLNLGIAPPLASDCPNENINLSSFPFQLGCNDNPVNMTARNENSVPGLPATMRTQLPHG
                        ********************************* **:***********************

Rr_PETALOSA_wt          SMVATTEPPLMSSMTSNFFPIHMERAMEKSMDANSFPNWAWQMQGLNDGATPVPLFSAAA
Rc_XP_024186592         SMVASTEPPLMNSMNSNFFPIHMERAMEKSMDANSFPNWAWQMQVLNDGATPVPLFSAAA
                        ****:******.**.***************************** ***************

Rr_PETALOSA_wt          SSGFPSSTTESSAPIPPLHFPNTSFLQRHFSPSFTNKTPHFYCRS
Rc_XP_024186592         SSGFPSSTTESSAPIPPLHFPNTSFLQRHFSPSFTNKTPHFYCRS
                        *********************************************
```

Figure S3. Sequence alignment of *PET* alleles from different species. Sequences deduced from targeted sequencing were: *D. caryophyllus* wild type and *pet* CDS (A) and peptide (B), *P. hybrida* wild type and *pet* CDS (C) and peptide (D) and *R. rugosa* wild type and *pet* CDS (E) and peptide (F). Sequence comparison between the wild-type peptide sequence of *R. chinensis* and *R. rugosa* PET (G).

Figure S4

A

```
KU096996_phBOB               CAATAGAGAAGGGCTTGGAAGTTGACACCTCATCCAAATGGATGCGGCAA
Peaxi162Scf00472g00069.1     CAATAGAGAAGGGCTTGGAAGTTGACACCTTATCCAAATGGATGCGGCAA
Phybrida_wt_cDNA             CAATAGAGAAGGGCTTGGAAGTTGACACCTTATCCAAATGGATGCGGCAA
Phybrida_pet_cDNA            CAATAGAGAAGGGCTTGGAAGTTGACACtgtaacaactaaattcgcgtca
                             ****************************   *:*.*.::..:*   * .*

KU096996_phBOB               GACCAGAATTCTTATGGTGGAAGTCCTACAGCTCCATTCTTCTCTACTGC
Peaxi162Scf00472g00069.1     GACCAGAATTCTTATGGTGGAAGTCCTACAGCTCCATTCTTCTCTACTGC
Phybrida_wt_cDNA             GACCAGAATTCTTATGGTGGAAGTCCTACAGCTCCATTCTTCTCTACTGC
Phybrida_pet_cDNA            agaaaataaatatcgagacaagaaaatattgcaacaatatattttattta
                             ....*.:*:: :*. .*: .*..:..** :**:.**:*.*: * ** * .

KU096996_phBOB               AGCATCATCAGGATTCGTAAATTCAGCAGTCAGTGCACCTTCAGCTGTCG
Peaxi162Scf00472g00069.1     AGCATCATCAGGATTCGTAAATTCAGCAATCAGTGGACCTTCAGCTGTTG
Phybrida_wt_cDNA             AGCATCATCAGGATTCGTAAATTCAGCAATCAGTGGACCTTCAGCTGTTG
Phybrida_pet_cDNA            tcaaaatatgagtgttacaatctctatgattcctctgattcgtcttttca
                             : .*:.:: ..*: * . **: **:. ..* . *  .. *  :  * * .

KU096996_phBOB               TTCATCAATTGCACTTTCCTAGTAGAGCACTACCTTACCACTATTCACCA
Peaxi162Scf00472g00069.1     TTCATCAACTGCACTTTCCTAGTAGAGCACTACCTTACCACTATTCACCA
Phybrida_wt_cDNA             TTCATCAACTGCACTTTCCTAGTAGAGCACTACCTTATCACTATTCACCA
Phybrida_pet_cDNA            aactataacttcaagggcttttgagcttgatcttgaagtttggatttgga
                             ::*:: ** * **.   * *:  **.  ..*.   :*  :  .:* :  *

KU096996_phBOB               TCACTCACAAATGAAACCTTCACATTATTATTGCAGGAGCTGAAATAG
Peaxi162Scf00472g00069.1     TCCCTCACAAATGAAACCTTCACATTATTATTGCAGGAGCTGAAATAG
Phybrida_wt_cDNA             TCCCTCACAAATGAAACCTTCACATTATTATTGCAGGAGCTGAAATAG
Phybrida_pet_cDNA            tttggatttggctttggatttgatcttgatcttgacttgatgagttca
                             *    .: :..  ::. .** ..: *: :: *  *  :*.***.:*..
```

B

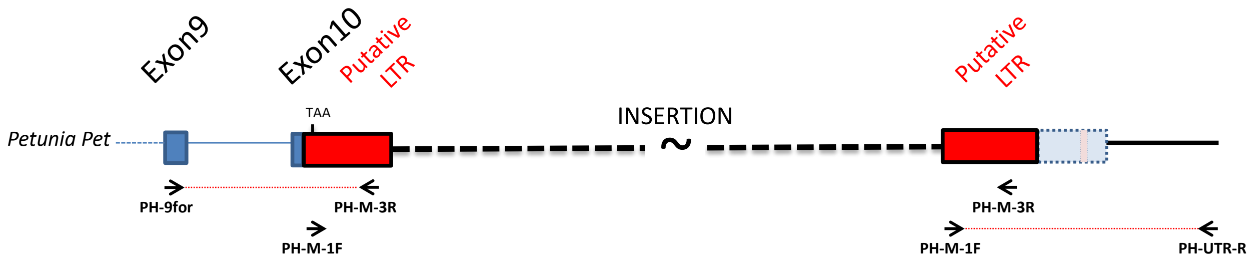

>putative_pet_LTR

**TGTAACAACTAAATTCGCGTCAAGAAAATAAATATCGAGACAAGAAAATATTGCAACAATATATTTTATTTATCAAAATATGAGTGTTACAATCTCTATGATTCCTCTGATTCGTCTTTTCAAACTA
TAACTTCAAGGGCTTTTGAGCTTGATCTTGAAGTTTGGATTTGGATTTGGATTTGGCTTTGGATTTGATCTTGATCTTGACTTGATGAGTTCAAGAGCTTGGAGCTTTATCTTGAACTTGGTCTTGAT
CTTGAACTTGTACTTGGATTCAAGGGCTTGAAGCTTGATCTTGAATCTTCGTCTTGATATCTAGAACTTGTAGAGAAATATTTGAGTGCTTGCAGCTTTTGTAGAAATCTCTTGCTTTGATCCACGA
GCTCTTTCTTGCTTCTTGTTATGGATTCTTGCTTCTGATTTCTTTTATTTTTGAAATGATGGATTTGACCCCTTTATATAGATGTGGGGTGGAGTAGCCACCAAGAAACCCTGACGGGCCTTGGCCCG
TGAACCGGCCAATCAGATTTAAGCAATGACTAGGCCGTATTTGATTGGCCATAACATGTCACATGTCCACATGGCGTATTTTCATTGGTCTTGGGGTTTGACTTGGATTGCCACATCATTTGACGTG
TGGCATGATCCAATTGGCTATTTTATTTGACTTGGCGCCACGTCACTTGCCATGTGGCACATTTTTGGGCCTTTAGGAAAACTTATATCCATTGGGCCATAAGCTAGTTGGGCTAGCCTAGCAAGAT
TGGACTTTTATTATATTTAAATAACTAGCCCAATTATATTAGCCCATAGCACTTATTTGGACCAATATATTTTTAATTTAATATATAGCCATTTTATTTCATGGATTTTGATCCAATAAAATTTACTTG
TCTACAGACAC**

Figure S4

C

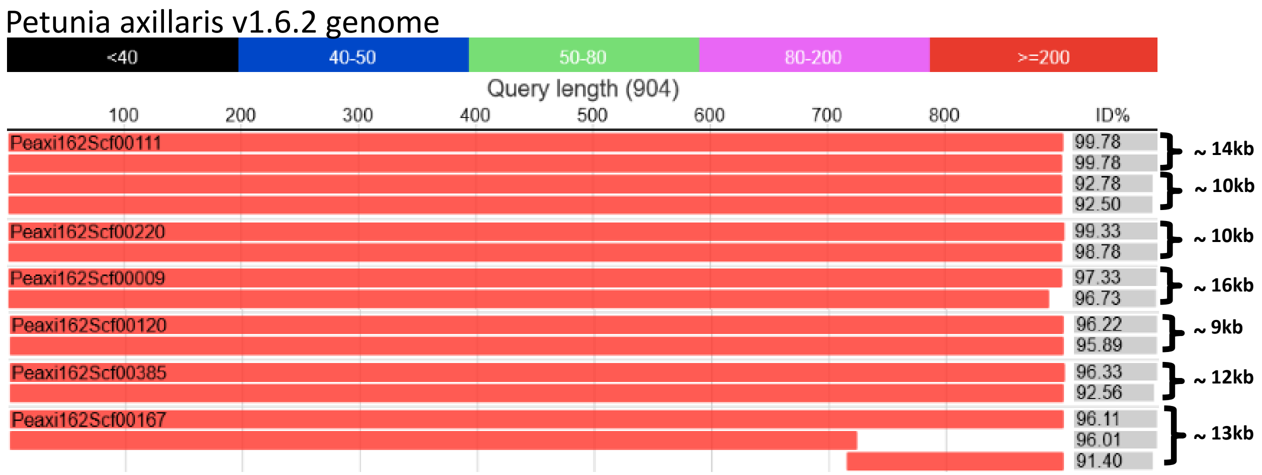

Figure S4: Analysis of the petunia *pet* allele. (A) Sequence alignment of the 10th CDS exon of wild type and *pet* alleles from DF petunia and the corresponding sequence of *BOB* (KU096996) and the *P. axillaris* hortologue *Peaxil62Scf00472g00069.1*. The diverging sequence in *pet* is indicated in italics, miR172 binding sites in grey and stop codons in bold; (B) PCR products were obtained from the genomic *pet* sequence both with primers PH-9F/PH-M-3R and PH-M-1F/PH-UTR-R, while no primer combination resulted in an amplification product of the whole insertion. The resulting putative LTR consensus sequence was used in BLAST searches against the *P. axillaris* genome: (C) Several paired BLAST hits were obtained in different scaffolds, separated by 9-16Kb of DNA.

Figure S5

A

```
                                                      miR172 binding site
NtomBEN         GATCGCCCTCTGCTCTGGAATGGAGTGAACACCAGTGTCTTTCCCACATTTAAGGGAACA
NtBENb          GATCGCCCTCTGCTCTGGAATGGAGTGAACACCAGTGTCTTTCCCACATTTAAGGGAACA
NtBENa          GATCGCCCTCTGCTCTGGAATGGAGTGAACACCAGTGTCTTTCCCACATTTAAGGGAACA
NtBOB           CAGCACCCCCTCCATTGGAATGGATTGAATGGGAATCTCTTTCCCACATTTAAGGGAACA
NtomBOB         CAGCACCCCCTCCATTGGAATGGATTGAATGGGAATCTCTTTCCCACATTTAAGGGAACA
                 * *.*** ** *: ********* **** .  *.* ***********************

NtomBEN         GCAATAGGGAAAGGTATGGAAGTTGATTCTTCACCAAATTGGACTCGGCAAGACCAACAT
NtBENb          GCAATAGGGAAGGGTATGGAAGTTGATTCTTCACCAAATTGGGCGCGGCAAGACCAACAT
NtBENa          GCAATAGGGAAAGGTATGGAAGTTGATTCTTCACCAAATTGGACTCGGCAAGACCAACAT
NtBOB           TCAATAGAGAAGGGCTTGGAAGTTGACACCTCTTTAAAATGGACGCAGCAAGGCCAAAAT
NtomBOB         TCAATAGAGAAGGGCTTGGAAGTTGACACCTCTTTAAAATGGACGCAGCAAGGCCAAAAT
                 ******.***.** :********** :* **:  ***:***.* *.*****.****.**

                                                     miR172 binding site
NtomBEN         CCTTATGGTGGGAGTCCTTCAATGCCACTCTTCTCTACTGCAGCATCATCAGGATTCGCT
NtBENb          CCTTATGGTGGGAGTCCTTCAGTGCCACTCTTCTCTACTGCAGCATCATCAGGATTCGCT
NtBENa          CCTTATGGTGGGAGTCCTTCAATGCCACTCTTCTCTACTGCAGCATCATCAGGATTCGCT
NtBOB           ACTTATGGTGGGAGACCTACAGTGTGATTCTTCTCTACCGCAGCATCATCAGGATTCGGA
NtomBOB         ACTTATGGTGGGAGACCTACAGTGTGATTCTTCTCTACCGCAGCATCATCAGGATTCGGA
                .*************:***:**.**  * ********** ******************* :

NtomBEN         AATTCAACCTCAGCTGCTGCTCATCAGCACCACTTTTCTACTGGGCCATTACCTTATCAT
NtBENb          AATTCAACCTCAGCCGCTGCTCATCAACACCACTTTTCTACTGGGTCATTACCTTATCAT
NtBENa          AATTCAACCTCAGCTGCTGCTCATCAGCACCACTTTTCTACTGGGCCATTACCTTATCAT
NtBOB           AATTCAGCAATCACT-------ATCAACTGCACTTTGGAAGTGAAGCTTTATCTTACCAC
NtomBOB         AATTCAGCAATCACT-------ATCAACTGCACTTTGGAAGTGAAGCTTTATCTTACCAC
                ******.*.: ..*        ****.*: ******  :* **.. *:*** **** **

NtomBEN         CATTCGCCATCACTTGCCAATATGAACTTTGCTCATTATTACTGCAGGAGTTGAAGTAGT
NtBENb          CATTCACCATCACTTGCCAATATGAACTTTGCACATTATTACTGCAGAAGCTGAAGTAGT
NtBENa          CATTCGCCATCACTTGCCAATATGAACTTTGCTCATTATTACTGCAGGAGTTGAAGTAGT
NtBOB           CACTCACCATCCCTGACCAACATGAATCTTTCACATCATTATTGTAGTAGCTGAAATTAG
NtomBOB         CACTCACCATCCCTGACCAACATGAATCTTTCACATCATTATTGTAGTAGCTGAAATTAG
                ** **.*****.** .**** *****  ** *:*** **** ** ** ** ****.*:.

NtomBEN         TTGCTATTTGATTAAAAAAAACAACCACCCCATATCTTTTCTTCCTCTGCTCTGGGAAAG
NtBENb          TTGCTATTTG--ACAAAAAAACAACCACCCGATATCTTTTCTTCCTCTGCTCTGGGAAAG
NtBENa          TTGCTATTTGATTAAAAAAAACAACCACCCCATATCTTTTCTTCCTCTGCTCTGGGAAAG
NtBOB           CTGG--CCAGTAAATGCAAAACAACCACCCAGATATCTTTCTTCCTTCGCTCAAG--ATG
NtomBOB         CTGG--CCAGTAAATGCAAAACAACCACCCAGATATCTTTCTTCCTTCGCTCAAG--ATG
                 **     :*  :.:..************* .:::  *********  ****:.*  *:*
```

Figure S5

B

```
Sequence ID: SRA:SRR8115234.10109496.1

Query  2    TTTCCCACATTTAAGGGAACATCAATAGAGAAGGGCTTGGAAGTTGACACCTCTTTAAAA  61
            ||||||||||||||||||||||||||||||||||||||||||||||||||||||||||||
Sbjct  125  TTTCCCACATTTAAGGGAACATCAATAGAGAAGGGCTTGGAAGTTGACACCTCTTTAAAA  66

Query  62   TGGACGCAGCAAGGCCAAAATACTTATGGTGGGAGACCTACAGTGTGATTCTTCTCTACC  121
            ||||||||||||||||||||||||||||||||||||||||||||||||||||||||||||
Sbjct  65   TGGACGCAGCAAGGCCAAAATACTTATGGTGGGAGACCTACAGTGTGATTCTTCTCTACC  6

Query  122  GCAGC  126
            |||||
Sbjct  5    GCAGC  1
```

```
Sequence ID: SRA:SRR8115234.8822533.2

Query  21   CATCAATAGAGAAGGGCTTGGAAGTTGACACCTCTTTAAAATGGACGCAGCAAGGCCAAA  80
            ||||||||||||||||||||||||||||||||||||||||||||||||||||||||||||
Sbjct  1    CATCAATAGAGAAGGGCTTGGAAGTTGACACCTCTTTAAAATGGACGCAGCAAGGCCAAA  60

Query  81   ATACTTATGGTGGGAGACCTACAGTGTGATTCTTCTCTACCGCAGCATCATCAGGATTCG  140
            ||||||||||||||||||||||||||||||||||||||||||||||||||||||||||||
Sbjct  61   ATACTTATGGTGGGAGACCTACAGTGTGATTCTTCTCTACCGCAGCATCATCAGGATTCG  120

Query  141  GAAAT  145
            |||||
Sbjct  121  GAAAT  125
```

```
Sequence ID: SRA:SRR8115234.8822533.2

Query  25   AATAGAGAAGGGCTTGGAAGTTGACACCTCTTTAAAATGGACGCAGCAAGGCCAAAATAC  84
            ||||||||||||||||||||||||||||||||||||||||||||||||||||||||||||
Sbjct  125  AATAGAGAAGGGCTTGGAAGTTGACACCTCTTTAAAATGGACGCAGCAAGGCCAAAATAC  66

Query  85   TTATGGTGGGAGACCTACAGTGTGATTCTTCTCTACCGCAGCATCATCAGGATTCGGAAA  144
            ||||||||||||||||||||||||||||||||||||||||||||||||||||||||||||
Sbjct  65   TTATGGTGGGAGACCTACAGTGTGATTCTTCTCTACCGCAGCATCATCAGGATTCGGAAA  6

Query  145  TTCAG  149
            |||||
Sbjct  5    TTCAG  1
```

```
Sequence ID: SRA:SRR8115234.7753861.2

Query  37   CTTGGAAGTTGACACCTCTTTAAAATGGACGCAGCAAGGCCAAAATACTTATGGTGGGAG  96
            ||||||||||||||||||||||||||||||||||||||||||||||||||||||||||||
Sbjct  1    CTTGGAAGTTGACACCTCTTTAAAATGGACGCAGCAAGGCCAAAATACTTATGGTGGGAG  60

Query  97   ACCTACAGTGTGATTCTTCTCTACCGCAGCATCATCAGGATTCGGAAATTCAGCAATCAC  156
            ||||||||||||||||||||||||||||||||||||||||||||||||||||||||||||
Sbjct  61   ACCTACAGTGTGATTCTTCTCTACCGCAGCATCATCAGGATTCGGAAATTCAGCAATCAC  120

Query  157  TATCA  161
            |||||
Sbjct  121  TATCA  125
```

Figure S5

C

Sequence ID: SRA:SRR2106531.61788434.2

```
Query  42    AAGTTGACACCTCTTTAAAATGGACGCAGCAAGGCCAAAATACTTATGGTGGGAGACCTA  101
             ||||||||||||||||||||||||||||||||||||||||||||||||||||||||||||
Sbjct  100   AAGTTGACACCTCTTTAAAATGGACGCAGCAAGGCCAAAATACTTATGGTGGGAGACCTA  41

Query  102   CAGTGTGATTCTTCTCTACCGCAGCATCATCAGGATTCGG  141
             ||||||||||||||||||||||||||||||||||||||||
Sbjct  40    CAGTGTGATTCTTCTCTACCGCAGCATCATCAGGATTCGG  1
```

Sequence ID: SRA:SRR2106531.58035425.1

```
Query  43    AGTTGACACCTCTTTAAAATGGACGCAGCAAGGCCAAAATACTTATGGTGGGAGACCTAC  102
             ||||||||||||||||||||||||||||||||||||||||||||||||||||||||||||
Sbjct  100   AGTTGACACCTCTTTAAAATGGACGCAGCAAGGCCAAAATACTTATGGTGGGAGACCTAC  41

Query  103   AGTGTGATTCTTCTCTACCGCAGCATCATCAGGATTCGGA  142
             ||||||||||||||||||||||||||||||||||||||||
Sbjct  40    AGTGTGATTCTTCTCTACCGCAGCATCATCAGGATTCGGA  1
```

D

```
PhBOB           MFDLNLSCDDVPEESDQLNELSVTQMENSRTSSTIGDDDNSCSDHMSNCY
NtBOB           MFDLNLSSEDVPVESG----TSNSSIVNMETSNSTAGDDEYEESGNSDFV
                *******.:*** **.     * :.: * .**.: ..**:  ..  *:

PhBOB           TFDILKANRKESENLGSFVTKELLPLSLNDQSMVLAQQQRLQVIKKSRRG
NtBOB           LKELFPLVSGEAELSQQQCLDLSANCGASNEQRIVISPQRRSQVKKNRRG
                  :::     *:*   .   .     . .::. :: . ** . :**.***

PhBOB           PRSRSSQYRGVTFYRRTGRWESHIWDGGKQVYLGGFDTAHAAARAYDRAA
NtBOB           PRSRSSQYRGVTFYRRTGRWESHIWDGGKQVYLGGFDTAHTAARAYDRAA
                ****************************************:*********

PhBOB           IKFRGFDADINFNVSDYEEDLKQMKNFSKEEFLHILRRQSTGFSRGSSKF
NtBOB           IKFRGLDADINFNISDYEDDLKQMNNFSNEEFVHILRRQSNGFSRGSSKY
                *****:*******:****:*****:***:***:*******.********:

PhBOB           RGVTLHKCGRWEARMGQLLGKKYIYLGLFDSEIEAARAYDKAAIKSNGRE
NtBOB           RGVTLHKCGRWEARMGQLLGKKYIYLGLFDSEIEAARAYDKAAIQSSGRE
                ********************************************:*.***

PhBOB           AVTNFELSTYEGVLSSETADTGGTSHNLDLRLGISPSSCADNQHGNTSQM
NtBOB           AVTNFEISTYEGVLSSEADIRG-TGHNLDLNLGISPSSYDDNQHGNTSQT
                ******:**********:   * *.*****.*******  *********

PhBOB           GISQCRPGSNGLPEHR--EVLSSASTTPRSMLLHGQHMLDQHPLHWNGPN
NtBOB           GNFQGQHGSNGLTEHHRKVLNSASTTLRSELLLQGQHVLTQHPLHWNGLN
                *  * : *****.**:   : *:::*   .:**:***:* ******** *

                                                          (miR172)
PhBOB           DNLFPTFKGTAIEKGLEVDTLSKWMRQDQNSYGGSPTAPFFSTAASSGFV
NtBOB           GNLFPTFKGTSIEKGLEVDTSLKWTQQGQNTYGGRPTV------------
                .*********:*********  ** :*.**:*** **.

PhBOB           NSAISGPSAVVHQLHFPSRALPYHYSPSLTNETFTLLLQELK
NtBOB           ------------------------------------------
```

E

```
                                     PAM
XP_016499635    TTCTCTACTGCAGCATCATCAGGATTCGCTAAT NtBENb
XP_016482517    TTCTCTACTGCAGCATCATCAGGATTCGCTAAT NtBENa
XP_016502850    TTCTCTACCGCAGCATCATCAGGATTCGGAAAT NtBOB   (miR172 binding site in 3'UTRs)
XP_016501408    TTTACTGCTGCAGCATCATCAGGATTCTCAACT NtTOE1
XP_016495732    TTTACTGCTGCAGCATCATCAGGATTCTCAACT NtTOE1
XP_016432764    TTCTACACTGCAGCATCATCAGGATTCTTAACT NtTOE1
XP_016460979    TTCTACACTGCAGCATCATCAGGATTCTTAACT NtTOE1
XP_016475254    TTTTCTTCTGCAGCATCATCAGGATTCTCTACC NtTOE2
XP_016454719    TCTTCTTCTGCAGCATCATCAGGATTCTCTACC NtTOE2
XP_016491201    GCAACTGCTGCAGCATCATCAGGATTCCCTCAG NtROB
XP_016483586    GCAACTGCTGCAGCATCATCAGGATTCCCCCAG NtROB
XP_016482629    GCAACTGCTGCAGCATCATCAGGATTCCCCCAG NtROB
XP_016459840    GCAACTGCTGCAGCATCATCAGGATTCCCTCAG NtROB
```

Figure S5. Analysis of the tobacco *PET* alleles (A) Alignments of 3' end of cDNAs encoding the two *N. tabacum* BEN XP_016499635 (NtBENb, XM_016644149.1) and XP_016482517 (NtBENa, XM_016627031.1), *N. tabacum* BOB XP_016502850 (NtBOB, XM_016647364.1) and *N.tomentosiformis* BEN XP_009606014 (NtomBEN, XM_009607719.2) and BOB XP_018630941 (NtBOB, XM_018775425.1). Putative stop codons are highlighted in red. (B) BLAST hits of *NtBOB* inRNA-Seq SRA SRX4941542 (RNA-Seq of *N. tabacum* leaf and flower tissues), showing the position of the STOP codon and miR172 binding site in the transcript. (C) BLAST hits of *NtBOB* in (RNA-Seq SRA SRX1100400: RNA-Seq of *N. tomentosiformis* leaf tissue), showing the position of the STOP codon and miR172 binding site in the transcript. (D) Alignment of the putative *N. tabacum* BOB peptide and the *P .hybrida* BOB peptide. (E) Sequence comparison of the DNA region targeted for genome editing (in black) specific for both *NtBENs* and the corresponding portions of other *N. tabacum* euAP2. Sequence corresponding to the miR172 binding site is underlined and the PAM site (AGG) is indicated. The sequence upstream the PAM site includes 13 nucleotides which are part of the well conserved miRNA binding site and 7 nucleotides granting target site specificity.

Figure S6

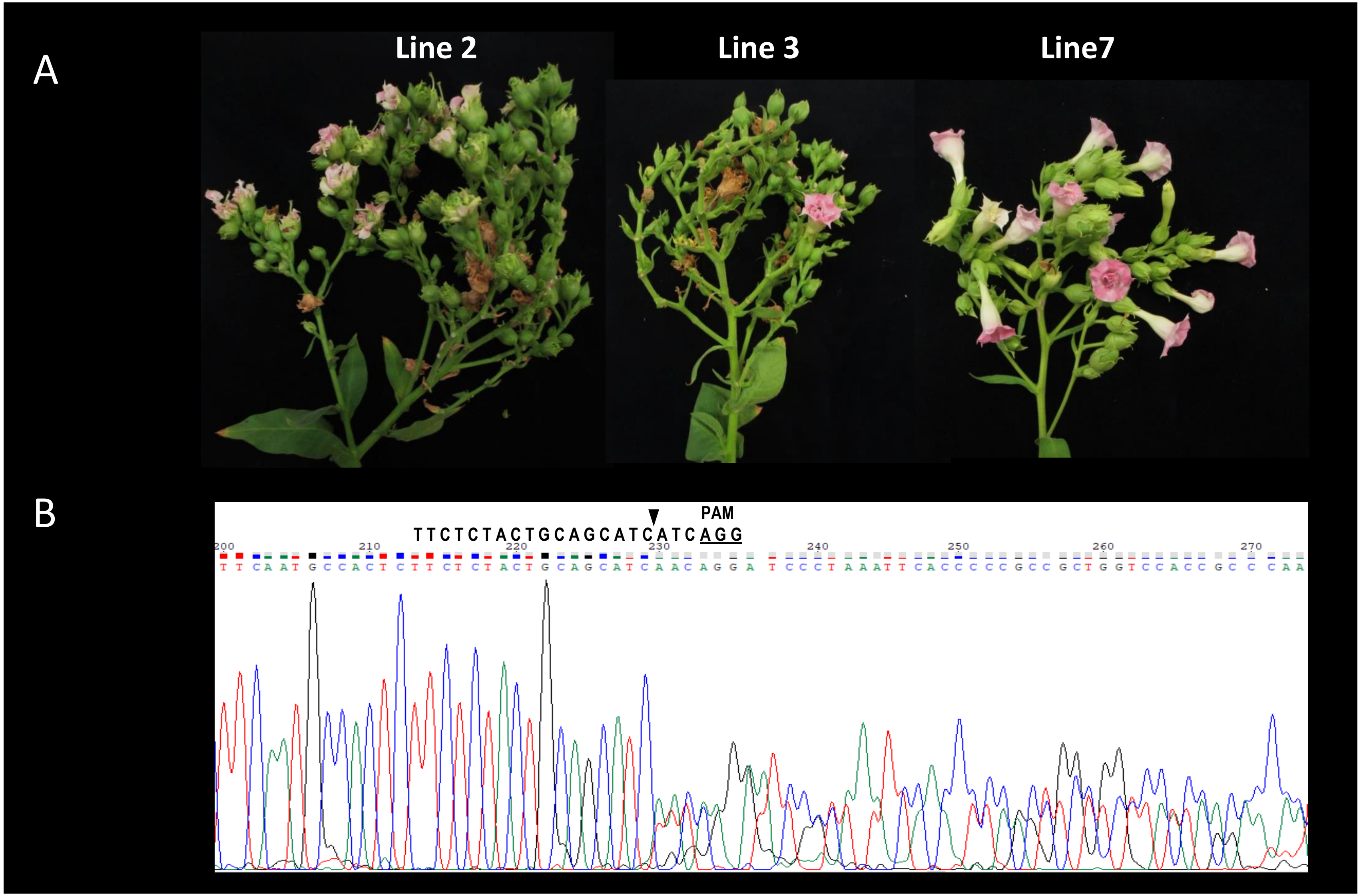

Figure S6. T_0 tobacco lines transformed with the CrispR-Cas9 construct. (A) Example of the range of DF phenotypes in three T_0 lines (2, 3 and 7). (B) Example of Sanger sequencing of the *NtBEN* (XP_016482517) gene in line 7, revealing lesions in the miR172 target site.

Figure S7

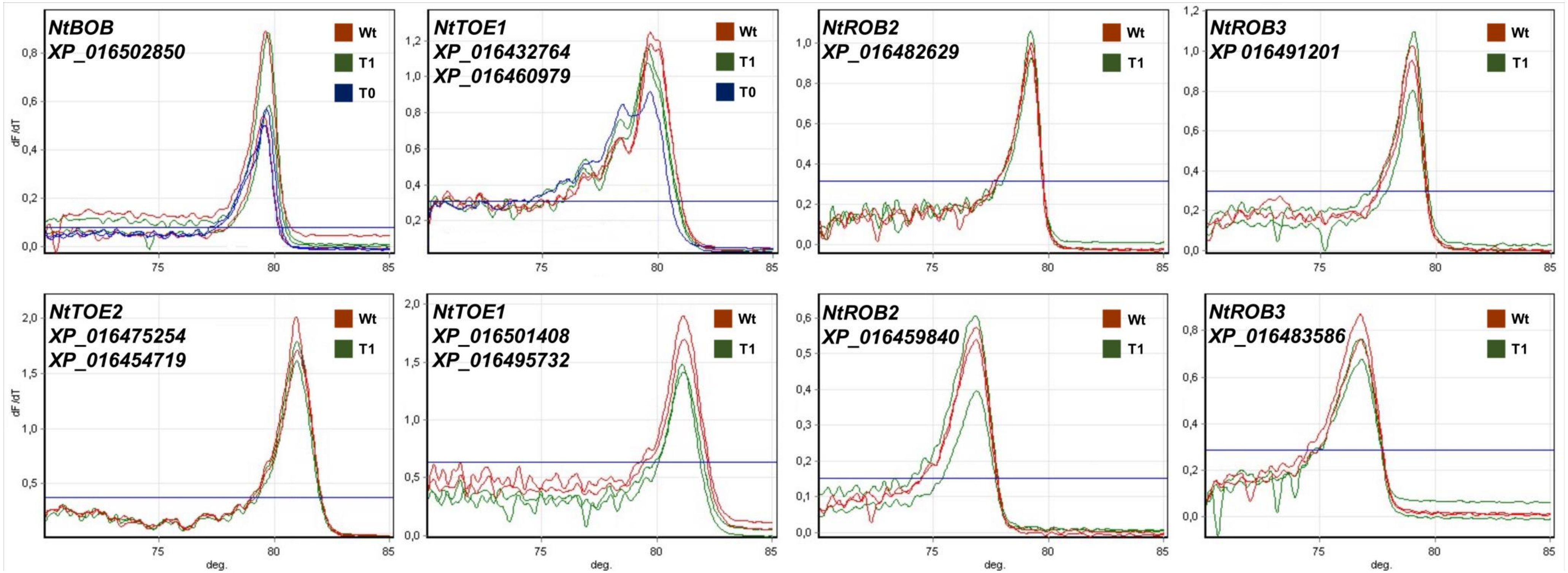

Figure S7. High-Resolution Melting Analysis for detection of potential off-target edited alleles of tobacco TOE-type genes. Differences in melting temperatures among samples were assessed through the evaluation of first derivative plot (dF/dT), comparing T1 line IV and wild-type (Wt) tobacco. For *TOE1* (XP_016432764 and XP_016460979) and *NtBOB* (XP_016502850) also T0 line 7 is shown: an editing event is present in *NtBOB* in T0 but not in T1. In each plot two replicates per sample are shown.

Figure S8

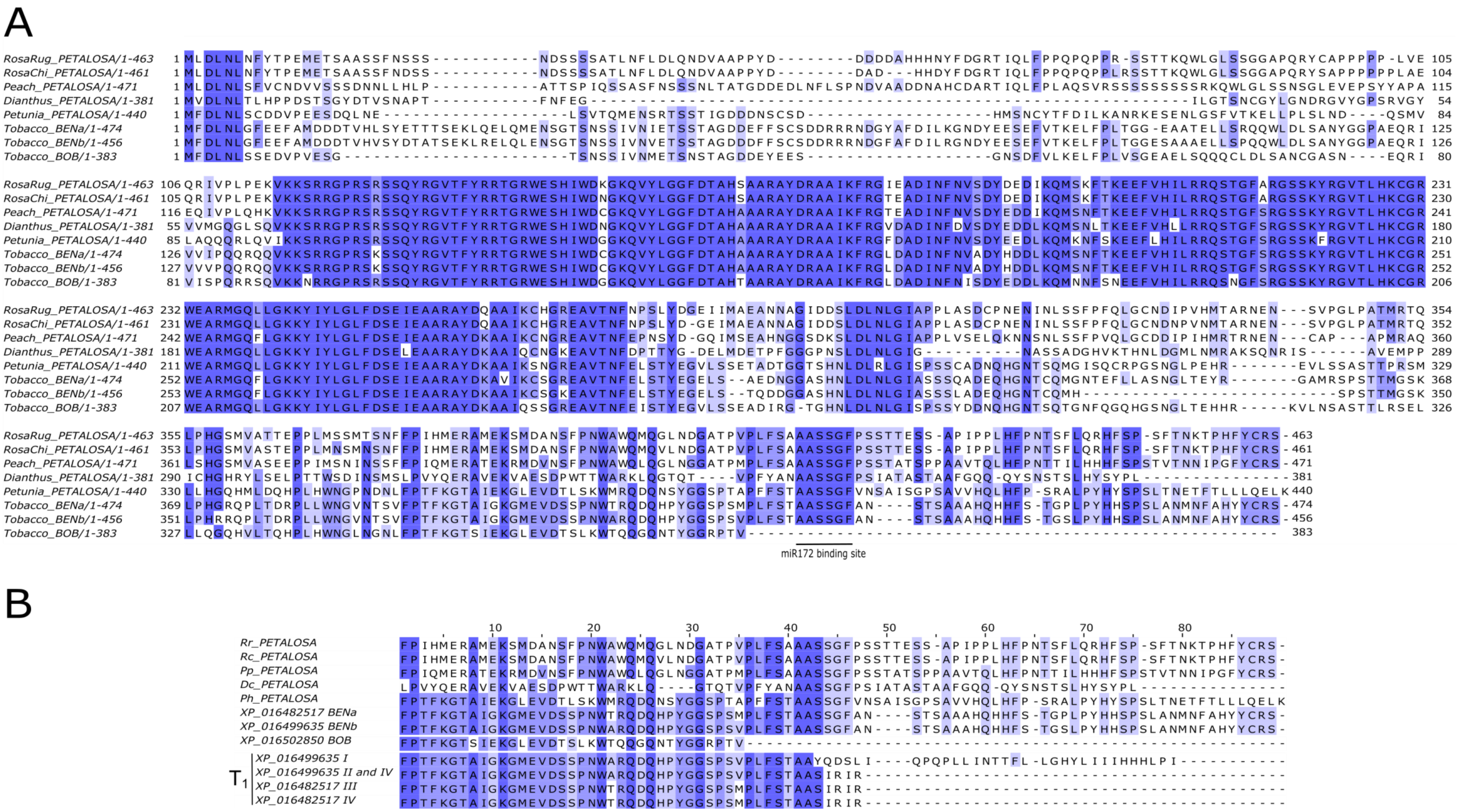

Figure S8. Degree of conservation of PET amino acid sequences and effect of CrispR-Cas9-induced mutations on protein sequences. (A) Alignment of deduced amino acid sequences of PET proteins from *R. rugosa*, *R. chinensis*, *P. persica*, *D. caryophyllus*, *P. hybrida* and three putative *N. tabacum* PET. Blue shading indicates degree of conservation of amino acid residues. (B) Alignment of C-ter portions of the same proteins and deduced PET in T1 gene-edited tobacco plants.

Table S1

Primer name	Sequence 5' - 3'	Use
CA-1f	CACTACAATGCACCTTCCTCCC	PCR Dianthus
CA-9f	CCTCCGATTTGTCACGGTC	PCR Dianthus
CA-1r	GTGCAAGTCATATCAGGTTACGG	PCR Dianthus
CA-4r	CGATTCAGACTATGAACAC	PCR Dianthus
PH-1f	GATTCTACAATTTACTGCCCCAG	PCR Petunia
PH-9f	GAATGGACCGAATGACAATC	PCR Petunia
PH-M-1r	CTTGACGCGAATTTAGTTGTTAC	PCR Petunia
PH-M-3r	TTCAAGATCAAGACCAAGTTC	PCR Petunia
PH-10r	TAGGAAAGTGCAGTTGATG	PCR Petunia
PH-UTRr	GGCATTGGTAATATCCGGTG	PCR Petunia
RO-8f	CTTGATCTAAACTTGGGAATTGC	PCR *Rosa rugosa*
RO-8f2	GGCTTCTGACGGTCCGAATG	Genome Walking *Rosa rugosa*
RO-1f	GGCCACAATTACTAGTACTAG	PCR *Rosa rugosa*
RR-3UTRr	ACAGTTACGTAGCCTGTTAC	PCR *Rosa rugosa*
B26	GACTCGAGTCGACATCGTTTTTTTTTTTTTTTTTT	3' RACE
B25	GACTCGAGTCGACATCG	3' RACE and Adapter Genome Walking
B25_TaqI_adapterF	**CG**CGATGTCGACTCGAGTC	Adapter Genome Walking
TOB-CRISPR_FOR	GATTGTTCTCTACTGCAGCATCATC	Guide CrispR Tobacco
TOB-CRISPR_REV	AAACGATGATGCTGCAGTAGAGAAC	Guide CrispR Tobacco
NTBEN_016482517_F	TTATCTGATATTGATAGGAG	Sequencing Tobacco
NTBEN_016499635_F	TCCAGTTTTCTTATCTGATGGA	Sequencing Tobacco
NTBEN_SEQR	GCCTCATTCCTAAGAAAACTAG	Sequencing Tobacco
LBfor	CTTTGCCCTCGGACGAGTGC	Screening CrispR Transgene
LBrev	GCGTATATGCTCCGCATTGG	Screening CrispR Transgene
RBfor	TCGAGCAGATCAGCGAGTTC	Screening CrispR Transgene
RBrev	ACCACTTTGTACAAGAAAGC	Screening CrispR Transgene
HRMA_NtBOB_f	GGACGCAGCAAGGCCAAAATACTT	High-Resolution Melting Analysis
HRMA_NtBOB_r	CTTCCAAAGTGCAGTTGATAGTGA	High-Resolution Melting Analysis
HRMA_NtBEN_f	ACATCCTTATGGTGGGAGTCCTTC	High-Resolution Melting Analysis
HRMA_NtBEN_r	GAGCAGCAGCTGAGGTTGAATTAG	High-Resolution Melting Analysis
HRMA_NtTOE1a_f	TCAGGTTGATACAGCCCCAGTGTC	High-Resolution Melting Analysis
HRMA_ NtTOE1a_r	ATGCTGAAGAAGTGATAAAAGCAG	High-Resolution Melting Analysis
HRMA_NtTOE2_f	AGTCACTCCAGTGCCCGTGTTTTC	High-Resolution Melting Analysis
HRMA_ NtTOE2_r	GGAGTCGTAGTTGGTTGCTTGGC	High-Resolution Melting Analysis
HRMA_NtTOE1b_f	CAATGTCAATGTTTACTGCTGCAG	High-Resolution Melting Analysis
HRMA_ NtTOE1b_r	GTATTCGGGTTGGGACCAGACAT	High-Resolution Melting Analysis
HRMA_NtROB1_f	GAAATTTCGCTGTCAACAAGCAAT	High-Resolution Melting Analysis
HRMA_ NtROB1_r	TTGAGGCCTTACTATCTGCTGGG	High-Resolution Melting Analysis
HRMA_NtROB3a_f	AATAATATCCCTCCTCCTCAGCAA	High-Resolution Melting Analysis
HRMA_ NtROB3a_r	CATGAGAGTATTTTTCTGAGACCG	High-Resolution Melting Analysis
HRMA_NtROB3b_f	TTCTCAAGAATGGTACCCTAATAA	High-Resolution Melting Analysis
HRMA_ NtROB3b_r	CAATTTTGAGGTCTTACTATCTGC	High-Resolution Melting Analysis
HRMA_NtROB2a_f	TTCTCAAGAATGGTACCCTAATAA	High-Resolution Melting Analysis
HRMA_ NtROB2a_r	CAATTTTGAGGTCTTACTATCTGC	High-Resolution Melting Analysis
HRMA_NtROB2b_f	CGTTTTCATCAAGTGATAGTCAAC	High-Resolution Melting Analysis
HRMA_ NtROB2b_r	GTTTTGATGTCTTAATATCTGCTG	High-Resolution Melting Analysis

Table S1. List of primers used in this study.

Table S2

A

Species	Variety	Flower **S**ingle or **D**ouble	*pet* CA-9f/CA-1r	wild type CA-9f/CA-4r
Dianthus caryophyllus	Pixie Star	**S**	-	+
Dianthus caryophyllus	Olivia	**S**	-	+
Dianthus caryophyllus	Neon Star	**S**	-	+
Dianthus caryophyllus	India Star	**S**	-	+
Dianthus caryophyllus	Starburst	**D**	+	+
Dianthus caryophyllus	Coral Reef	**D**	+	+
Dianthus caryophyllus	Lady in Red	**D**	+	+
Dianthus caryophyllus	Widecombe Fair	**D**	+	+
Dianthus caryophyllus	Mrs Sinkins	**D**	+	+
Dianthus caryophyllus	Raspberry Sundae	**D**	+	+
Dianthus caryophyllus	Sissy	**D**	+	+
Dianthus caryophyllus	Passion	**D**	+	+
Dianthus caryophyllus	Cranmere Pool	**D**	+	+
Dianthus caryophyllus	Purple Wings	**D**	+	+
Dianthus caryophyllus	SuperTrouper series	**D**	+	+
Dianthus caryophyllus	Francesco	**D**	+	-
Dianthus caryophyllus	Viper Wine	**D**	+	-
Dianthus gratianopolitanus	Jane Austen	**D**	+	+
Dianthus gratianopolitanus	La Bourboule Alba	**S**	-	+
Dianthus gratianopolitanus	Badenia	**S**	-	+
Dianthus chinensis	Floral Lace picotee	**S**	-	+
Dianthus barbatus	Sweet William White	**S**	-	+
Dianthus barbatus	Sweet William Purple	**S**	-	+
Dianthus deltoides	Flashing lights	**S**	-	+
Dianthus superbus	Primadonna	**S**	-	+

B

R. rugosa Variety	Flower **S**ingle or **D**ouble	*pet* band	wild type band
typica	**S**	-	+
Nyveldt's White	**S**	-	+
Hansa	**D**	+	+
Blanc Double de Coubert	**D**	+	+
Pierette	**D**	+	+
Red Foxi	**D**	+	+
Gufo della Neve	**D**	+	+

TableS2. (A) PCR analysis of 11 single-flower and 14 DF *Dianthus* varieties. The primer combinations indicated are specific for pet (CA-9f/CA-1r) or the wild-type allele (CA-9f/CA-4r). (B) PCR analysis of 2 single-flower and 5 DF *R. rugosa* varieties using primers RO-8f e RR-3UTRr. "**+**" indicates the presence of the PCR band.
